# Injured cardiac targeting magnetic nanovesicles for mRNA treatment of myocardial infarction

**DOI:** 10.7150/thno.124754

**Published:** 2026-01-21

**Authors:** Dasom Mun, Ji-Young Kang, Malgeum Park, Gyeongseo Yoo, Jaewoong Lee, Nuri Yun, Boyoung Joung

**Affiliations:** 1 Division of Cardiology, Yonsei University College of Medicine, Seoul 03722, Republic of Korea.; 2 GNTPharma Science and Technology Center for Health, GNTPharma Incheon 21983, Republic of Korea.

**Keywords:** nanovesicles, lipid nanoparticles, targeted delivery, myocardial infarction, mRNA therapy

## Abstract

**Rationale:** Inflammation and myocardial remodeling are major contributors to the progression of cardiac diseases. mRNA-based therapeutics have emerged as a promising modality for cardiovascular intervention; however, their clinical translation remains constrained by challenges in achieving efficient and spatially precise delivery to diseased cardiac tissue, particularly following myocardial injury. To address this unmet need, a dual-active magnetic nanocarrier was engineered for targeted mRNA delivery to damaged cardiovascular tissue.

**Methods:** The interleukin-10 anti-inflammatory cytokine mRNA (*IL-10* mRNA) was encapsulated in lipid nanoparticles, which were fused with nanovesicles derived from mesenchymal stem cells (NVs) and functionalized with cardiac-targeting peptides (T peptides) to form *IL-10* mRNA-loaded T-NVs (*m10*@T-NVs). Magnetic nanoparticles (MNPs) were conjugated with azide-modified antibodies against CD63 and myosin light chain 3 (MLC3), which are overexpressed in damaged myocardial tissue *via* click chemistry, to enable targeted delivery to injured cardiac tissue. Subsequently, the *m10*@T-NVs were combined with functionalized MNPs *via* CD63 interactions to form *m10*@T-MNVs.

**Results:** *m10*@T-MNVs were developed and characterized, confirming the functionalization of NVs and MNPs. Under guided of an external magnetic field, *m10*@T-MNVs exhibited a 4.5-fold increase in accumulation in H_2_O_2_-induced injured cardiomyocytes and damaged cardiac regions, achieving significantly higher delivery efficiency. In a mouse model of myocardial infarction (MI), administration of *m10*@T-MNVs enhanced intramyocardial IL-10 mRNA expression and cytokine production. This led to the polarization of macrophages toward an M2 anti-inflammatory phenotype, mitigation of tissue injury, reduced apoptosis, attenuation of fibrosis, and suppression of pathological myocardial remodeling.

**Conclusions:** Dual-active targeting of injured cardiac tissue using magnetic nanocarriers constitutes a promising therapeutic strategy for cardiovascular diseases by addressing key challenges associated with tissue-selective mRNA delivery in the injured myocardium.

## Introduction

Messenger RNA (mRNA) therapeutics have demonstrated the potential for genome editing, immunotherapy, and protein replacement therapy 1. Building on this therapeutic potential, lipid nanoparticle (LNP)-based delivery systems have achieved great success, particularly in vaccine applications, and continue to show exceptional promise for a wide range of clinical uses 2. LNPs typically comprise ionizable lipids, cholesterol, phospholipids, and poly(ethylene glycol) (PEG)-lipids 3. At low pH, the ionizable lipids acquire a positive charge that facilitates complexation with negatively charged mRNA during nanoparticle formation 4. However, LNP lipid composition, likely due to the ionizable cationic lipid or other positively charged residuals, can lead to nonspecific interactions with cell membranes or immune cells, contributing to toxicity and immunogenicity 5. Recent studies have attempted to incorporate biomimetic elements, such as cell membrane-derived lipids, into LNPs to enhance their biocompatibility and immune evasion and mitigate these issues 6. Fusion of cell membrane-derived lipids with LNPs yields a biomimetic coating that attenuates the surface charge and reduces LNP-induced cytotoxicity 7. The efficient delivery of therapeutic mRNAs to non-hepatic tissues remains challenging with current LNPs, primarily because of their preferential accumulation in the liver, which is influenced by the ionizable properties of lipid components and rapid clearance by Kupffer cells 8. Membrane components that mimic the composition of natural cells have been introduced to reduce immune responses, enhance biocompatibility, and limit hepatic uptake to mitigate this issue 9. Although selective organ-targeting LNPs have recently been developed to favor accumulation in organs, such as the lungs, liver, and spleen, systematic efforts to achieve passive or active targeting to the heart remain underexplored 10.

Given the complex cellular heterogeneity and physiological barriers of the myocardium, precise delivery to the cardiac tissue, and more importantly, to specific cardiac cell types, is essential for improving therapeutic efficacy following myocardial infarction (MI) 11. Recent studies in the field of cardiac research have demonstrated that LNP-mediated mRNA delivery provides improved transfection efficiency across diverse cardiac cell types, including fibroblasts, endothelial cells, and epicardial cells, compared to non-nanoparticle-based formulations 12. In parallel, intracoronary administration has been reported to enhance the myocardial distribution of mRNA-LNPs more broadly than intravenous or intramyocardial routes in both healthy and injured hearts, although its ability to achieve cell-specific delivery remains limited 13. While these findings provide valuable insights into the delivery efficiency and biodistribution, further investigations are needed to develop strategies that can achieve more selective and therapeutically relevant targeting within the diseased myocardium.

**Figure 1:**
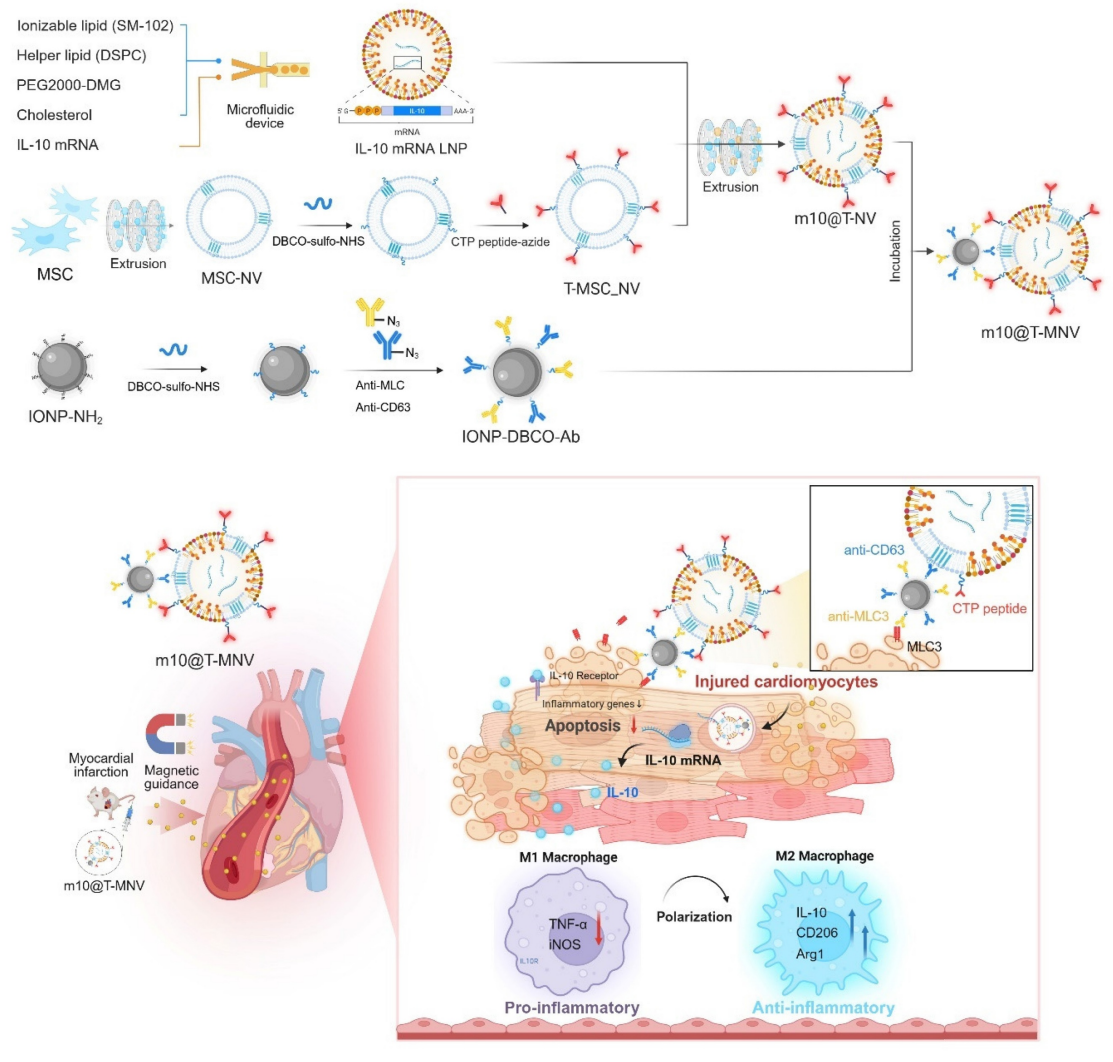
** Injured-cardiac-targeting magnetic nanovesicles for mRNA-based treatment of myocardial infarction.** Mesenchymal stem cell-derived nanovesicles (MSC-NVs) were modified with cardiac-targeting peptides (T peptides) and co-extruded with *IL-10* mRNA-loaded lipid nanoparticles (*IL-10* mRNA/LNPs). Magnetic nanoparticles conjugated with anti-CD63 and anti-MLC3 antibodies were incorporated to generate *m10*@T-MNVs. Upon intravenous injection and magnetic guidance, *m10*@T-MNVs target the injured myocardium, deliver *IL-10* mRNA, and reprogram infiltrating macrophages toward an anti-inflammatory M2 phenotype, thereby reducing apoptosis, inflammation, and fibrosis after MI.

We developed a hybrid nanocarrier system, *m10*@T-MNVs, to deliver interleukin-10 anti-inflammatory cytokine mRNA (*IL-10* mRNA) selectively to the injured myocardium. The platform integrates magnetic nanoparticles (MNPs) with MSC-derived nanovesicles (MSC-NVs) and allows modular surface functionalization through click chemistry. MLC3 was chosen as a lesion-level targeting motif because cytoskeletal MLC isoforms become exposed upon sarcolemmal rupture after MI, enabling antibody access despite their intracellular origin, and prior studies have shown successful MLC3-directed nanoparticle targeting *in vivo* 14, 15. Accordingly, MNPs were conjugated with azide-functionalized anti-Cluster of Differentiation 63 (CD63) and anti-MLC3 antibodies to promote injury-specific accumulation. To introduce cardiac-level specificity, NVs were modified with azide-conjugated cardiac-targeting peptides (T peptides). These peptides were originally identified through *in vivo* phage display screening and are known to preferentially bind myocardial tissue *in vivo* 16, 17. The hybrid nanocarrier system integrates a dual-ligand approach, where CD63 facilitates the interaction between MNPs and NVs, while T peptides and MLC3 enhance the targeting of injured cardiac tissue (Figure 1). This platform also enabled the encapsulation of *IL-10* mRNA within lipid nanoparticles (LNPs), thereby preserving mRNA stability and bioactivity for *in vivo* applications. In this study, we evaluated the targeting efficiency, therapeutic efficacy, and safety of this hybrid system in both *in vitro* and *in vivo* models of myocardial infarction.

## Materials and Methods

### Preparation of IL-10 mRNA

The mRNA encoding IL-10 was synthesized by Bioneer (Daejeon, Korea) using a T7 polymerase-based *in vitro* transcription system. The transcript contained a 5′ CleanCap (Cap 1) structure, a 3′ poly(A) tail and consisted of unmodified nucleotides. As a negative control, an uncapped version of the *IL-10* mRNA, synthesized under identical conditions but without CleanCap addition, was prepared by Bioneer (Daejeon, Korea). Purification was performed *via* silica column-purification (Bioneer AccuPrep® Universal RNA Extraction Kit). mRNA quality and integrity were assessed by denaturing agarose gel electrophoresis (1% formaldehyde-containing gel). Samples were loaded onto a 1% agarose gel, and electrophoresis was conducted at 100 V for 30 min in TAE buffer. Band images were quantified with ImageJ software (National Institutes of Health, Bethesda, MD, USA).

### Preparation of IL-10 mRNA-Loaded LNPs and characterization

LNPs encapsulating *IL-10* mRNA were prepared using a microfluidic mixing approach. The lipid components included SM-102, DSPC, cholesterol, and PEG2000-DMG in a molar ratio of 50:10:38.5:1.5, respectively. Lipids were dissolved in ethanol, and *IL-10* mRNA was diluted in Tris buffer (pH 7.4). The aqueous and organic phases were mixed at a 1:3 (v/v) ratio using a microfluidic mixer (LinaPrep™, RNApharm, Korea), maintaining an N/P ratio of 1:4. The resulting LNP dispersion was dialyzed against Tris-buffered saline (TBS, pH 7.4) for 24 h to remove ethanol and unencapsulated components. The size and PDI of the IL-10 loaded LNPs were characterized using dynamic light scattering (DLS, Zetasizer Nano-ZS, Malvern Panalytical, Ltd., Malvern, UK). To confirm the presence of mRNA encapsulated within the lipid nanoparticles, the particles were lysed using Triton X-100, and the released RNA was analyzed by 1% TBE agarose gel electrophoresis.

### RiboGreen assay

Encapsulation efficiency was assessed *via* RiboGreen RNA assay (Thermo Fisher Scientific, USA) according to the manufacturer's instructions. Briefly, samples were diluted in TE buffer and mixed with RiboGreen reagent to measure unencapsulated RNA fluorescence. For total RNA quantification, parallel samples were treated with 0.1% Triton X-100 to lyse the LNPs and release encapsulated RNA. Fluorescence intensity was measured using a Varioskan LUX microplate reader (Thermo Fisher Scientific, USA) at excitation/emission wavelengths of 500/525 nm. Encapsulation efficiency was calculated as: EE (%) = (mRNA concentration in lysed sample - mRNA concentration in non-lysed sample)/mRNA concentration in lysed sample × 100.

### Preparation of MSC-NVs

Human bone marrow mesenchymal stem cells (MSC, ATCC, Manassas, VA, USA) were maintained in low-glucose Dulbecco's modified Eagle's medium (LM 007-07, Welgene, Gyeongsan, Korea) containing 10% fetal bovine serum (US-FBS-500, Welgene) and 1% penicillin-streptomycin (10378016, Gibco), and incubated at 37 °C under a humidified atmosphere with 5% CO₂. MSC-NVs were generated from MSCs (ATCC, USA) according to the serial extrusion established in previous studies using an extrusion method 18. As soon as the cultured MSCs reached ~90% confluence in 150 mm^2^ culture dishes, the cells were detached from the dishes by a cell scraper and centrifuged at 300 × *g* for 5 min. The samples were subjected to sonication at 1000 V with 20% power for 6 cycles, where each cycle consisted of a 4-s pulse followed by a 2-s pause. After sonication, the samples were incubated on ice for 2 min to minimize thermal effects. The sonication and cooling process were then repeated once under the same conditions. To obtain the MSC-NVs, the MSCs were suspended in phosphate buffered saline (PBS), and serially extruded nine times through 5-μm, 1-μm, 0.4-μm, and 0.2-μm pore sizes polycarbonate membrane filters using a mini extruder (Avanti Polar Liquids, Birmingham, USA).

For density-gradient ultracentrifugation, 0.5 ml of 10% iodixanol (Optiprep, Axis-Shild, Oslo, Norway) was layered on top of 0.5 ml of 50% iodixanol in an ultracentrifuge tube (Himac CS120GXL). The supernatants were overlaid on top of the 10% iodixanol, and centrifuged at 100,000 × g for 2 h at 4 °C using a Himac CS120GXL microcentrifuge (Hitachi, Tokyo, Japan). To remove residual iodixanol and further purify the MSC-NVs, The NVs-containing fraction was collected and subsequently centrifuged at 100,000 × g for 1 h at 4 °C. The pellet was gently resuspended in PBS and subjected to two washing steps using an Amicon Ultra-4 centrifugal filter unit (UFC8010; Millipore Corporation, Bedford, MA, USA). The MSC-NVs were resuspended in PBS and were stored at -80 °C, until use.

### Surface functionalization of MSC-NVs with T peptides

To conjugate MSC-NVs with heart-targeting moiety T peptides, DBCO-sulfo-N-hydroxysuccinimidyl ester (NHS) was covalently introduced to amine-groups present on MSC-NVs *via* click chemistry. Specifically, DBCO-sulfo-NHS (3 μM; Peptron Co., Daejeon, South Korea) was allowed to react with MSC-NVs at a concentration of 0.5 mg mL⁻¹ in phosphate-buffered saline while gently rotated at ambient temperature for 4 h. Unreacted DBCO-NHS was removed by three consecutive washes employing centrifugal filter units with a 30-kDa molecular weight cutoff (Millipore). The resulting DBCO-modified nanovesicles were subsequently incubated with azide-bearing T peptide (T; APWHLSSQYSRT), a scrambled peptide control (S; ARPLEHGSDAT), or Cy5.5 fluorophore (Hallandale Beach, FL, USA) at a final concentration of 0.3 μM in PBS under continuous mixing at 4 °C for 12 h.

### Assembly of *m10*@T-NVs

Assembly of *m10*@T-NVs: To construct cardiac-targeted hybrid nanovesicles (*m10*@T-NVs), T-MSC-NVs were co-extruded with *IL-10* mRNA/LNPs at a 1:1 ratio (w/w) at room temperature. The mixture was passed 11 times through a 100-nm polycarbonate membrane using a mini-extruder (#610020, Avanti Polar Lipids, Inc.). This assembly enabled preferential accumulation in infarcted cardiac tissue and enhanced *IL-10* mRNA delivery efficiency.

### DBCO-IONP conjugation

Amine (-NH₂)-functionalized iron oxide nanoparticles were prepared at a concentration of 0.5 mg mL⁻¹ (IONPs; Sigma-Aldrich; Merck KGaA, Darmstadt, Germany, molecular weight: 231.53 g/mol). For the conjugation of DBCO-sulfo-NHS to IONPs, the prepared iron oxide nanoparticle solution was mixed with 2.16 μmol of DBCO-sulfo-NHS (Peptron Co., Daejeon, South Korea) at a 1:1 molar ratio and incubated on a rotating mixer at room temperature for 4 h. The mixture was then centrifuged at 10,000 × *g* for 10 min to remove unconjugated DBCO-sulfo-NHS.

### Azide modification of antibodies

For click chemistry, anti-Myosin light chain 3 (MLC3; ab233220, Abcam, 0.2 mg mL^-1^), and anti-CD63 (clone E-12, sc-365604, Santa Cruz Biotechnology, 0.5 mg mL^-1^) antibodies were site-specifically modified on the Fc regions of the heavy chain with an azide using the Site Click™ Antibody Azido Modification Kit (Thermo Fisher Scientific, USA). Briefly, 50 μg of anti-MLC3 antibody, and 50 μg of anti-CD63 antibody were loaded onto an antibody concentrator and centrifuged for 6 min at 5,000 × *g*. The flow-through was discarded and 450 μL of antibody preparation buffer was added and centrifuged for 8 min at 5,000 × *g*. Galactosidase was then added to the antibody solution and incubated overnight at 37 °C to remove the galactose residues. Further, the azide group was enzymatically attached to the carbohydrate modified antibody. Therefore, Tris buffer, buffer additive and GalT (Y289L) enzyme along with the carbohydrate modified antibody were added to a tube containing UDP-GalNAz and incubated overnight at 30 °C. To remove excess UDP-GalNAz, the azide-modified antibody was washed with 1 mL of Tris buffer and centrifuged for 10 min at 1200*g*.

### IONP surface functionalization with antibody

Subsequently, 0.5 mg mL^-1^ DBCO-IONP were incubated with 17.5 μg of azide-modified antibodies (1 mg mL^-1^) overnight at 4 °C with rotation to facilitate conjugation through click chemistry between the DBCO and azide (-N₃) moieties established in previous studies 19. To remove unconjugated antibodies, the mixture was centrifuged at 10,000 × *g* for 10 min. After antibody conjugation, antibody-bound IONPs were collected by centrifugation, and the supernatant containing unbound antibodies was recovered for quantification. The concentration of unbound antibodies was determined by absorbance at 280 nm using a standard curve generated from the corresponding unconjugated antibody. The amount of surface-bound antibodies was calculated by subtracting the unbound fraction from the initial antibody input and expressed as micrograms of antibody per milligram of IONPs.

### Preparation and characterization of *m10*@T-MNVs

To verify the isolation quality and purity of *m10*@T-MNVs, particle size and morphology were evaluated by transmission electron microscopy (TEM), nanoparticle tracking analysis (NTA), and dynamic light scattering (DLS). For TEM observation, specimens were deposited onto a Formvar-carbon-coated grids (Leica Microsystems, Vienna, Austria). Samples were negatively stained with 2% uranyl acetate for 15 s and subsequently examined by transmission electron microscopy (TEM; JEM-1011, JEOL Ltd., Tokyo, Japan). For SEM analysis, samples were fixed in Karnovsky's fixative for 24 h, washed in 0.1 M phosphate buffer, post-fixed with 1% OsO₄ for 2 h, dehydrated through graded ethanol, critically point-dried (EM CPD300), carbon-coated (EM ACE600), and imaged using a field-emission SEM (MERLIN, ZEISS). EDS elemental mapping was performed with a Bruker XFlash® 5060 FlatQUAD detector at 15 kV, ×2000 magnification, and 300 s acquisition time using ESPRIT 2.1. FT-IR spectra of the samples were recorded using an FT-IR spectrometer (INVENIO, Bruker, Germany). Dried samples were scanned from 400-4000 cm⁻¹ with a resolution of 4 cm⁻¹, and 32 scans were averaged for each measurement. Background spectra were collected prior to sample acquisition and automatically subtracted. The size distribution and concentration of MNVs were measured using a NanoSight LM10 instrument (Malvern, UK) and NTA software version 2.3 (Malvern, UK). The dynamic light scattering (DLS) was performed by electrophoretic light scattering using an ELS-1000ZS analyzer (Otsuka Electronics, Osaka, Japan). To evaluate the storage stability of *m10*@T-MNVs, samples were stored at 4 °C and -80 °C, and the mean particle diameter was measured on Days 0, 7, and 14 using dynamic light scattering (ELS-1000ZS; Otsuka Electronics, Osaka, Japan).

### Magnetic responsiveness analysis

For *in vitro* experiments, a circular neodymium magnet (2 T) was positioned beneath the culture dish for 30 min. After exposure to an external magnet, fluorescence images were acquired using a confocal laser scanning microscope (LSM 710; Carl Zeiss, Jena, Germany).

### Cell culture and H_2_O_2_ treatment

H9C2 rat cardiomyocytes (ATCC, Manassas, VA, USA) were cultured in Dulbecco's modified Eagle medium (LM001-05, Welgene, Gyeongsan, Korea) supplemented with 10% FBS (US-FBS-500, Wellgene, Gyeongsan, Korea) and 1% penicillin-streptomycin (Cat. No. 10378016, Gibco, Grand Island, NY, USA) under humidified conditions at 37 °C with 5% CO₂. To simulate MI-like conditions, H9C2 cells were treated with 400 μM H₂O₂ (216763, Sigma-Aldrich) for 24 h. As a macrophage cell model, RAW264.7 murine macrophage cells (ATCC, Manassas, VA, USA) were cultured in DMEM (LM 007-07, Welgene) supplemented with 10% FBS (Wellgene), and 1% penicillin-streptomycin (Thermo Fisher Scientific). Cells were maintained at 37°C in a humidified atmosphere containing 5% CO₂. For LPS stimulation, cells were treated with 100 ng mL⁻¹ of lipopolysaccharide (L7895, Sigma-Aldrich) for 4 h to activate inflammatory responses. After 4 h, the cells were collected with a cell scraper, and the macrophage polarization was analysed. For Magnetic responsiveness analysis, after exposure to an external magnet, fluorescence intensity line profiles were extracted across the imaging field using ImageJ and plotted as a function of distance.

### Fluorescence labeling and immunocytochemistry

To confirm the hybrid structure, cells were treated with Cy5.5-labeled MSC-NVs and PKH67-labeled LNPs co-extruded to form hybrid MNVs, and incubated for 6 h at 37 °C. For cellular uptake studies, purified MNVs were fluorescently tagged with PKH67 green dye (PKH67GL, Sigma-Aldrich), whereas *IL-10* mRNA/LNPs were labeled using a PKH26-based red fluorescent dye (PKH26GL, Sigma-Aldrich). After incubation of MNVs (20 µg mL⁻¹), cells were washed three times with PBS, fixed with 4% paraformaldehyde, and stained with Alexa Fluor 488-conjugated troponin I antibody (sc-133117-AF488, Santa Cruz Biotechnology, Dallas, TX, USA) and DAPI for nuclear staining. Fluorescence signals were visualized using a confocal laser scanning microscope (LSM 710, Carl Zeiss, Jena, Germany). For immunocytochemistry, cells were fixed in 4% paraformaldehyde, followed by membrane permeabilization and blocking using DPBS supplemented with 0.3% Triton X-100 and 3% bovine serum albumin, with the procedure carried out for 1 h at ambient temperature. The samples were then incubated with the following primary antibodies: anti-phospho-histone H3 (PH3; 1:200, Invitrogen, PA5-17869) and anti-α-actinin (sc-17829, 1:200, Santa Cruz Biotechnology).

### Endocytic and endosomal escape analysis

H9C2 cells were incubated with PKH26-labeled *m10*@T-MNVs (50 µg mL⁻¹) for 4 h or 8 h at 37 °C. After incubation, cells were washed twice with cold PBS and stained with LysoTracker Green (75 nM, Invitrogen) for 30 min at 37 °C to label acidic endolysosomal compartments. To identify the cellular uptake mechanisms of *m10*@T-MNVs, H9C2 cells were pretreated for 30 min at 37 °C with the following inhibitors: EIPA (50 µM; macropinocytosis inhibitor), genistein (200 µM; caveolae-mediated endocytosis inhibitor), and wortmannin (100 nM; phagocytosis-related PI3K inhibitor). After pretreatment, cells were incubated with PKH26-labeled *m10*@T-MNVs (50 µg mL⁻¹) for 4 h under standard culture conditions. Cells were washed, fixed with 4% paraformaldehyde, and counterstained with DAPI. Confocal images were obtained using identical imaging parameters, and Pearson's correlation coefficients were determined to compare colocalization efficiency between treatment groups.

### Assessment of cell viability and cytotoxicity

Viability was measured using the Quanti-Max™ cell viability assay kit (QM2500, Biomax Inc.) according to the manufacturer's instructions. The medium was replaced with culture medium containing WST-8 reagent, and the cells were incubated in a CO_2_ chamber at 37 °C for 0.5 to 4 h. Following incubation, optical density was measured at 490 nm using a microplate spectrophotometer (VersaMax). Culture supernatants were harvested and aliquoted (10 μL) into 96-well plates, after which 100 μL of LDH reaction mixture was added. After incubation at 37 °C for 4 h, absorbance was recorded at 450 nm using the same microplate reader (VersaMax).

### Measurement of apoptosis

Apoptosis was assessed by detecting phosphatidylserine externalization and membrane integrity *via* flow cytometry using the FITC Annexin V Apoptosis Detection Kit II (556570, BD Biosciences, San Jose, CA, USA). Cells were enzymatically released from culture dishes with trypsin-EDTA (25300062, Gibco) and suspended in Annexin V binding buffer. The cell suspensions were then incubated with Annexin V-FITC and propidium iodide under light-protected conditions at room temperature. Fluorescence signals were acquired on a BD LSR II SORP flow cytometer, and the resulting data were analyzed using BD FACSDiva software.

### *In vitro* co-culture model for macrophage polarization

To investigate the paracrine immunomodulatory effects of *m10*@T-MNVs, an *in vitro* co-culture system was established using H9C2 cardiomyocytes (ATCC) and RAW264.7 mouse macrophages (ATCC). Transwell inserts with 0.4 μm pore size (Corning, CLS3450) were used to spatially separate the two cell types while allowing for cytokine diffusion.

### ELISA assay

To assess the expression levels of IL-10 protein in culture media and mouse tissue, ELISA was performed using a commercial mouse IL-10 ELISA kit (Thermo Fisher Scientific). Cell culture media and mouse tissue lysates were prepared according to the manufacturer's instructions, and 96-well plates pre-coated with IL-10 capture antibodies were used for cytokine detection. Absorbance was measured at 450 nm using a microplate reader, and IL-10 concentrations were calculated based on the standard curve.

### Western blot analysis

Protein lysates were obtained after solubilization of samples in RIPA buffer (ATTO, Tokyo, Japan) supplemented with protease and phosphatase inhibitor mixtures. The extracted proteins were resolved on SDS-PAGE gels and subsequently immobilized onto PVDF membranes (IPVH00010; Millipore). Membranes were briefly equilibrated with EveryBlot™ Blocking Buffer (Bio-Rad Laboratories Inc., Hercules, CA, USA) at room temperature for 10 min, followed by incubation at 4 °C with primary antibodies overnight. The following antibodies were used: anti-CD63 (E-12; 1:1000, sc-365604), anti-LAMP2 (1:1000, sc-18822), anti-CD81 (1:1000, sc-166029), and anti-GAPDH (1:1000, sc-166574) (all from Santa Cruz Biotechnology). GAPDH was included as a reference marker for protein loading. Signal detection was carried out using a LAS-4000 Mini chemiluminescence imaging system (FUJIFILM, Tokyo, Japan).

### Quantitative reverse transcription PCR

RNA was isolated from cultured cells and tissue specimens with the RNeasy Mini Kit (74104; Qiagen, Hilden, Germany). cDNA was generated from the extracted RNA using the High-Capacity cDNA Reverse Transcription Kit (Applied Biosystems, Darmstadt, Germany). Quantitative PCR amplification was performed using the PowerUp™ SYBR™ Green Master Mix (Applied Biosystems) on the QuantStudio™ 3 Real-Time PCR System (Thermo Fisher Scientific). All primer sequences were synthesized by Cosmogenetech (Seoul, Korea) and are provided in Table S2.

### Animal experiments

All animal experiments were reviewed and authorized by the Institutional Animal Care and Use Committee of Yonsei University College of Medicine (approval reference 2021-0152). The experimental procedures were performed in accordance with established guidelines for the care and use of laboratory animals issued by the U.S. National Institutes of Health (Publication No. 85-23, revised 1996). All *in vivo* studies were conducted at Yonsei University College of Medicine (Seoul, Republic of Korea). For the myocardial infarction mouse model, male C57BL/6 mice (8 weeks old; Orient Bio Inc., South Korea) were anesthetized using intraperitoneal injection of xylazine (Rompun®, 10 mg kg⁻¹) and tiltamine-zolazepam (Zoletil, 30 mg kg⁻¹) and mechanically ventilated following tracheal intubation using a small-animal ventilator (Harvard Apparatus Co., Millis, MA, USA). Myocardial infarction was generated by permanent ligation of the left anterior descending (LAD) coronary artery with a 7-0 polypropylene suture, placed 2-3 mm below the left atrium. Sham-operated mice underwent the same procedure without LAD ligation. After intravenous administration of the formulations, a circular neodymium magnet (2 T) was placed over the cardiac region for 30 min to promote magnetically guided localization 15, 20. For the fibrosis model, C57BL/6 male mice were implanted with Alzet® 1002 micro-osmotic pumps (Durect Corp., Cupertino, CA, USA) delivering Ang II at 2 mg kg⁻¹ day⁻¹. Sham-operated mice underwent identical surgical procedures without Ang II infusion. Animals were randomly allocated into four experimental groups for subsequent analysis. After intravenous administration of the formulations, a circular neodymium magnet (2 T) was placed over the cardiac region for 30 min to promote magnetically guided localization 15, 20.

### *In vivo* biodistribution

The nanovesicles were administered intravenously through the tail vein (300 μg MNVs, 15 mg kg⁻¹ MNVs per mouse). Animals were euthanized 4 h post-administration, after which major organs, including the heart, lungs, liver, spleen, and kidneys, were excised. Whole-organ biodistribution was evaluated using the IVIS® Spectrum *in vivo* imaging system (PerkinElmer, Waltham, MA, USA). For evaluation of cardiac localization, harvested hearts were sectioned and processed for immunofluorescence staining using antibodies against cardiac troponin I (cTNI; ab47003, Abcam, Cambridge, UK), vimentin (ab92547, Abcam), and von Willebrand factor (vWF; ab6994, Abcam), with DAPI applied for nuclear visualization. Intracellular localization of Cy5.5-labeled MNVs within myocardial tissue was examined by confocal laser scanning microscopy (LSM 710, Carl Zeiss, Jena, Germany). Fluorescence images were quantitatively analyzed using ImageJ software (NIH, USA) following ROI definition based on thresholded marker channels, and signal intensity was calculated as mean fluorescence after background correction.

### Retention assay

To assess the systemic persistence of *m10*@T-MNVs, mice were intravenously injected with Cy5.5-labeled *m10*@T-MNVs at a dose of 15 mg/kg MNVs per mouse. At different time points (e.g., 2, 4, 6, 24, 48 and 72 h post-injection), whole blood was collected into EDTA-coated tubes to prevent coagulation. Blood samples were centrifuged at 2,000 × g for 10 min at 4 °C to obtain plasma. The fluorescence intensity of the plasma was measured using the IVIS^®^ Spectrum *in vivo* imaging system (PerkinElmer, Waltham, MA, USA).

### Hemolysis assay

Mouse whole blood (100 µL) was centrifuged at 3000 rpm for 10 min to isolate red blood cells (RBCs). The RBCs were washed 3 times with 1 mL PBS to remove contaminants and resuspended in 1 mL PBS. A 200 µL aliquot of the RBC suspension was mixed with 1 mL saline containing the test sample (1 µg mL⁻¹). For the positive control, RBC lysis was induced using 0.1% Triton X-100. The suspensions were incubated at 37 °C for 1 h, followed by centrifugation at 3000 rpm for 10 min. The supernatants were collected, and hemoglobin release was quantified by measuring the optical density (OD) at 575 nm using a microplate reader (Varioskan LUX, Thermo Scientific, USA).

### Histological evaluation

For histological assessment, tissue samples were collected, preserved in 4% formaldehyde, and processed into paraffin blocks. Sections were stained with hematoxylin to visualize nuclei, briefly differentiated in acid-alcohol, and counterstained with eosin to highlight the cytoplasm. The stained slides were examined using an inverted microscope (Olympus). To assess fibrotic remodeling in infarcted myocardium, both Masson's trichrome and Sirius Red staining (ab245887, Abcam, Waltham, MA, USA) were performed. In Masson's trichrome staining, collagen-rich fibrotic regions appeared blue, while Sirius Red specifically stained collagen fibers red under bright-field illumination and exhibited birefringence under polarized light. Fibrosis was quantified as the ratio of collagen-stained area to the total left ventricular area using ImageJ software (NIH, USA). To evaluate apoptosis, a TUNEL (Terminal deoxynucleotidyl transferase dUTP nick end labeling) assay was conducted, according to the manufacturer's instructions. TUNEL-positive nuclei were counted in infarcted and peri-infarct regions, and apoptotic index was calculated as the percentage of TUNEL-positive nuclei relative to total nuclei per field.

### *In vivo* safety profile

To evaluate the systemic safety of *m10*@T-MNVs *in vivo*, mice received MNVs by intravenous administration. Blood samples were collected from individual animals, and serum was separated by centrifugation at 600 × g for 10 min. Serum biochemical parameters, including glutamic-pyruvic transaminase (GPT), glutamic-oxaloacetic transaminase (GOT), albumin (ALB), and alkaline phosphatase (ALP), were quantified using an automated clinical chemistry analyzer (DRI-Chem 4000i, Fuji, Minato, Japan).

### Proteomic profiling

Representative heart lysates from each subject were homogenized in a protein extraction buffer (iNtRON Biotechnology, Seoul, Korea). Proteomic profiling was performed at Macrogen (Seoul, Republic of Korea) using the Olink Target 96 Mouse Exploratory Panel (Olink Bioscience, Uppsala, Sweden), based on proximity extension assay technology 21, 22. Data acquisition and normalization were conducted using NPX Signature software, with results expressed as log₂-transformed NPX values.

### Immunofluorescence staining

For immunohistochemical observation, the tissues were dissected, fixed in 4% paraformaldehyde, followed by paraffin embedding and sectioning into 4-µm-thick slices. Histological analyses were performed using H&E staining to evaluate tissue pathology, Masson's trichrome (MT) for the identification of injured areas. The nuclei were labeled with hematoxylin. The required antibodies were as follows: anti-iNOS (ab15323, Abcam), anti-CD206 (ab64693, Abcam), anti-MLC3 (ab233220, Abcam), mouse anti-rabbit (sc-516250, Santa Cruz Biotechnology) was used for fluorescent secondary antibody, and the nuclei were stained using DAPI (62248, Thermo Fisher Scientific). The tissues were observed under an inverted microscope (Olympus, Japan) and Zeiss LSM 710 confocal microscope (Carl Zeiss, Jena, Germany).

### Echocardiography analysis

Left ventricular systolic function was evaluated based on quantitative echocardiographic parameters, including left ventricular ejection fraction (LVEF) and left ventricular fractional shortening (LVFS). LVEF (%) was calculated as the percentage change between end-diastolic and end-systolic ventricular volumes, expressed as (LVEDV - LVESV) / LVEDV × 100, where LVEDV and LVESV correspond to the left ventricular volumes measured at diastole and systole, respectively. In parallel, LVFS (%) was determined from changes in left ventricular internal diameter, using the formula (LVIDd - LVIDs) / LVIDd × 100, with LVIDd and LVIDs representing internal diameters during diastolic and systolic phases.

### Statistical analysis

Statistical comparisons between two groups were conducted using a two-tailed Student's t-test, while multiple group comparisons were analyzed *via* one-way ANOVA with Tukey's post hoc test for pairwise comparisons. Data are expressed as the mean ± standard deviation. Statistical significance was defined as a *p*-value of less than 0.05. All statistical analyses were carried out using GraphPad Prism software version 8.0 (GraphPad Software, San Diego, CA, USA).

## Results and Discussion

### Preparation and functionalization of the *m10*@T-NVs

Interleukin-10 (IL-10) is a central anti-inflammatory cytokine that modulates the immune response by regulating the duration of inflammation and promoting a prototypical M2-like macrophage phenotype 23, 24. Recent studies demonstrated that IL-10 constrains sphingolipid metabolism in macrophages, thereby limiting inflammation at the metabolic level 25. In the context of MI, IL-10 suppresses apoptotic injured cardiomyocyte death and reprograms activated cardiac macrophages toward a reparative M2 phenotype, thereby attenuating profibrotic signaling and subsequent diastolic dysfunction 26, 27. To overcome the short half-life of recombinant IL-10 protein in circulation and to achieve sustained cytokine expression, IL-10 was encoded as mRNA and delivered using LNPs. Accordingly, we formulated *IL-10* mRNA-LNP (*IL-10* mRNA/LNPs) *via* a microfluidic mixing strategy to enable prolonged cytokine expression. LNPs were prepared using an ionizable lipid composition of SM-102 (Heptadecan-9-yl 8-((2-hydroxyethyl)(6-oxo-6-(undecyloxy)hexyl)amino)octanoate), PEG2000-DMG (1-Monomethoxypolyethyleneglycol-2,3-dimyristylglycerol), DSPC (1,2-Distearoyl-sn-glycero-3-phosphocholine), and cholesterol in a molar ratio of 50:10:38.5:1.5. The nitrogen to phosphate (N/P) ratio was fixed at 1:4, and the lipids and mRNA were mixed at a 1:3 (v/v) ratio to achieve efficient encapsulation (Figure S1A). Agarose gel electrophoresis was performed to confirm the *IL-10* mRNA synthesis. The integrity of the *in vitro* transcribed *IL-10* mRNA was verified using electrophoretic analysis, which showed a distinct single band of the expected size, confirming successful synthesis (Figure S1B). Agarose gel electrophoresis was performed to evaluate the encapsulation efficiency of mRNA in LNPs. The lane without Triton X-100 showed no free mRNA, whereas the lane with Triton X-100 revealed a released mRNA band. This indicated the successful encapsulation of mRNA within the LNPs (Figure S1C).

To mitigate the inherent cytotoxicity of synthetic *IL-10* mRNA/LNPs, we incorporated a biologically derived membrane component to enhance biocompatibility. Mesenchymal stem cell (MSCs) secrete a broad range of anti-inflammatory factors and extracellular vesicles that contribute to cardiac repair by promoting angiogenesis, suppressing inflammatory responses, and attenuating adverse remodeling post-MI 28, 29. These cell-derived vesicles exhibit natural tropism toward injured tissues and are known to modulate macrophage polarization in the infarcted myocardium, thereby complementing the immunotherapeutic effects of IL-10. Given their intrinsic regenerative and immunomodulatory properties, MSCs were selected as the biomimetic membrane source. To exploit these beneficial properties, human MSCs were serially extruded through polycarbonate membranes and subsequently ultracentrifuged to isolate MSC-derived nanovesicles (MSC-NVs). To impart cardiac targeting capability, MSC-NVs were functionalized with dibenzocyclooctyne (DBCO)-conjugated T peptides *via* click chemistry to generate T-MSC-NVs and enable cardiac targeting. Subsequently, T-MSC-NVs were co-extruded with *IL-10* mRNA/LNPs to form membrane-integrated hybrid vesicles, facilitating preferential accumulation within the desired tissue to enhance targeted delivery of *IL-10* mRNA. To achieve this, the MSC-NVs and *IL-10* mRNA/LNPs were co-extruded to form *m10*@T-NVs (Figure S2A).

To evaluate the efficiency of T peptide conjugation to nanovesicle surfaces, we quantified T peptide loading on NVs using a fluorescence-labeled T peptide. Quantitative analysis of the fluorescence signal confirmed successful T peptide incorporation in both T-MSC-NVs and *m10*@T-NVs, indicating efficient conjugation *via* click chemistry and preservation of surface-bound targeting moieties during the extrusion process (Figure S2B). The morphology and size were assessed using transmission electron microscopy (TEM), which showed that MSC-NVs, T-MSC-NVs, *IL-10* mRNA/LNPs and *m10*@T-NVs exhibited characteristic cup-shaped morphologies, with diameters ranging from 150 to 250 nm (Figure S2C). Nanoparticle tracking analysis (NTA) confirmed that MSC-NVs, T-MSC-NVs, *IL-10* mRNA/LNPs, and *m10*@T-NVs exhibited comparable particle sizes, indicating that peptide conjugation and mRNA loading did not significantly alter the overall size distribution (Figure S2D and E). Zeta potential measurements of MSC-NVs, T-MSC-NVs, *IL-10* mRNA/LNPs, and *m10*@T-NVs demonstrated changes in the surface charge, indicating the successful modification of *m10*@T-NVs (Figure S2F). Protein expression analysis of markers CD63, CD81, and LAMP2 confirmed the presence of NV markers and the preservation of key vesicular protein signatures throughout the engineering process (Figure S2G). Agarose gel electrophoresis was performed with and without Triton X-100 treatment to verify the successful loading of *IL-10* mRNA into the *m10*@T-NVs. No mRNA signal was detected due to encapsulation in the absence of detergent (N). A distinct RNA band corresponding to *IL-10* mRNA appeared upon lysis with Triton X-100 (T), confirming the physical incorporation of mRNA into the *m10*@T-NVs (Figure S2H). To assess whether membrane extrusion affected mRNA loading, we compared the encapsulation efficiency of *IL-10* mRNA in LNPs before and after hybridization with T-MSC-NVs. Both *IL-10* mRNA/LNPs and *m10*@T-NVs exhibited high encapsulation efficiencies (>80%), indicating that the extrusion and membrane fusion process preserved the integrity of the mRNA encapsulation (Figure S2I). Together with the previous experiments, these results confirmed the successful fabrication of *m10*@T-NVs.

### Preparation of the *m10*@T-MNVs

Prior studies have demonstrated that magnetic nanoparticles substantially enhance local particle retention and tissue-level deposition under flow or shear conditions when guided by an external magnetic field 20. Motivated by these findings, we developed magnetic nanoparticles conjugated to antibodies to target injured cardiomyocytes using magnetic nanovesicles. Building upon the previously established *m10*@T-NV platform, we next incorporated a magnetic guidance system to further enhance myocardial targeting. This dual-active nanoplatform, termed *m10*@T-MNVs, integrates two complementary targeting modalities—biological (*via* peptide-functionalized MSC-NVs) and magnetic (*via* anti-MLC3 antibody-functionalized iron oxide nanoparticles)—to improve the delivery of *IL-10* mRNA to infarcted cardiac tissue. The overall design and composition are illustrated in Figure 1A.

As the magnetic component of the dual-active nanoplatform, iron oxide nanoparticles (IONPs) were selected and subsequently modified to enable antibody-mediated targeting. Amine-functionalized IONPs exhibited uniform spherical morphology and nanoscale size distribution, as confirmed using TEM and scanning electron microscopy (SEM) analyses (Figure S3A). Specifically, an anti-MLC3 antibody was used to enable selective binding to injured myocardial tissue, ensuring precise targeting of the injured cardiac region, whereas an anti-CD63 antibody was used to recognize human mesenchymal stem cell-derived nanovesicles (MSC-NVs), thereby facilitating their efficient attachment and delivery 14, 15. The anti-MLC3 and anti-CD63 antibodies were site-specifically modified on the heavy chain Fc regions with an azide group by transferase and UDP-N-azidoacetylgalactosamine (UDP-GalNAz). These antibodies were conjugated to IONP-DBCO *via* click chemistry to form antibody-conjugated IONPs (IONP-Ab). We synthesized IONP-NH_2_ and conjugated them sequentially with DBCO-sulfo-NHS and azide-functionalized antibodies (anti-CD63 and anti-MLC3), as illustrated in the schematic diagram (Figure S3B), to generate injured cardiomyocyte-targeting magnetic nanovesicles.

Based on the standard curve of the antibodies, 1 mg mL⁻¹ of modified IONPs contained an average of 74.2 ± 1.7 µg of surface-bound antibodies, confirming efficient conjugation (Figure S3C). The zeta potential measurements of IONPs, IONP-DBCOs, and IONP-Abs demonstrated changes in the surface charge, indicating successful modification and effective development of IONP-Abs (Figure S3D). Fourier transform infrared (FTIR) spectroscopy was performed at each modification step to confirm the surface functionalization of the IONPs. The FTIR spectrum of unmodified IONPs exhibited characteristic peaks at ~580 cm^-1^, corresponding to Fe-O bond stretching. Upon conjugation with DBCO-sulfo-NHS (IONP-DBCO), new absorption bands appeared near ~1100-1300 cm^-1^, attributable to C-O and C-N stretching, indicating successful DBCO incorporation. Further conjugation with antibodies introduced additional peaks near ~1650 cm^-1^ and ~1540 cm^-1^, corresponding to C=O and N-H amide I bands, characteristic of antibody binding (Figure S3E). To confirm the conjugation between azide-antibody and IONP-DBCO, fluorescence images showed that anti-CD63 and anti-MLC3 antibodies were co-localized with the Cy5.5-labeled IONPs (Figure S3F, G). The vesicles exhibited a controlled spatial arrangement under a magnetic field, confirming their magnetic responsiveness (Figure S3H, I). These results indicated that antibody-engineered IONPs possess both targeted binding capabilities and magnetic responsiveness, enabling efficient targeting of injured cardiac tissue. Collectively, these results indicated that the engineered IONP-Abs possess both cell-specific recognition and magnetic targeting capabilities, which are essential for subsequent cardiomyocyte-targeting applications.

### Engineering and characterization of targeted magnetic nanovesicles

Building on this platform, we constructed magnetically responsive hybrid nanovesicles and examined IONP-Ab:*m10*@T-NV ratios (0:1-2:1) to identify a stable formulation. This ratio-dependent screen revealed a shift from partial surface decoration to overloading-associated aggregation as IONP-Ab content increased (Figure S4A). Notably, the hydrodynamic size remained relatively stable up to the 1:1 ratio, whereas a clear increase in particle size was observed at ratios above 1:1, suggesting excessive IONP-Ab decoration on the nanovesicle surface (Figure S4B). Parallel cytotoxicity and cell viability analyses demonstrated that higher IONP-Ab ratios induced increased cytotoxicity and reduced viability, whereas the 1:1 formulation maintained favorable biocompatibility (Figure S4C, D). Taken together, these data indicate that the 1:1 IONP-Ab:*m10*@T-NV ratio represents an optimal balance between magnetic surface functionalization and physicochemical stability. Based on this optimized condition, extruded *m10*@T-NVs (0.5 mg mL⁻¹) were incubated with IONP-Abs (0.5 mg mL⁻¹) to generate *m10*@T-MNVs for subsequent experiments.

To confirm the successful magnetic functionalization of *m10*@T-MNVs with IONP-Abs and to assess their structural integrity, SEM was performed. SEM images of the *m10*@T-MNVs revealed a cup-shaped morphology, likely resulting from the surface-conjugated magnetic nanoparticles, which appeared clustered on one side of the vesicle owing to their magnetic properties, verifying the successful magnetic functionalization of the *m10*@T-MNVs with IONP-Ab (Figure S5A). Energy-dispersive X-ray spectroscopy analysis of the elemental composition of IONPs detected Fe, Si, C, and O, confirming the presence of Fe₃O₄ in the IONPs. The elemental composition of the nanoparticles suggested the presence of IONP and MSC-NV components, validating their integration with *IL-10* mRNA/LNPs and MSC-NVs within the particles (Figure S5B, C). The stepwise modification of the *m10*@T-NVs, IONP-Abs, and *m10*@T-MNVs was further validated using the FTIR spectra (Figure S5D). Upon conjugation with T peptides, new absorption bands appeared near ~1100-1300 cm^-1^ (C-O), and ~2100 cm^-1^ (C=N=N), indicating successful T peptides incorporation. Further conjugation with IONP-Abs introduced additional peaks near ~580 cm⁻¹ (Fe-O). These results confirmed the synthesis and functional characterization of *m10*@T-MNVs.

**Figure 2:**
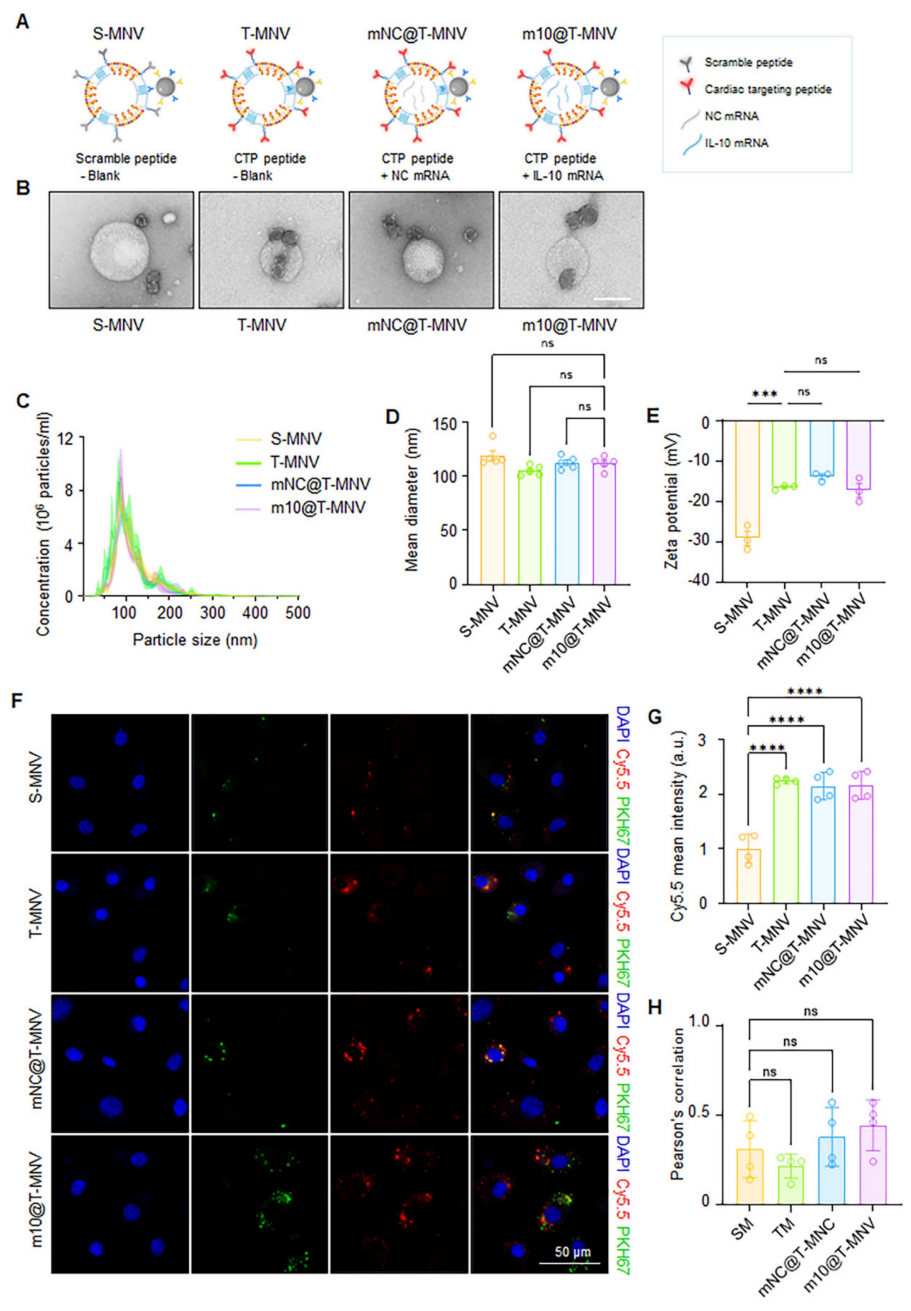
** Preparation and characterization of *IL-10* mRNA-loaded, magnetic cardiac targeting T-MNVs.** A) Schematic illustration of magnetic nanovesicle formulations: S-MNVs (scramble peptide, no mRNA), T-MNVs (T peptide, no mRNA), *mNC*@T-MNVs (T peptide with negative control mRNA), and *m10*@T-MNVs (T peptide with *IL-10* mRNA). B) Transmission electron microscopy images of respective nanovesicles. Scale bars, 100 nm. C) Nanoparticle tracking analysis of particle size distribution. D) Quantification of mean particle diameter. E) Zeta potential analysis of S-MNVs, T-MNVs, *mNC*@T-MNVs, and *m10*@T-MNVs. F) Representative confocal microscopy images showing intracellular uptake of S-MNVs, T-MNVs, *mNC*@T-MNVs, and *m10*@T-MNVs in H9C2 cardiomyocytes. MSC-NVs were labeled with Cy5.5 (red), *IL-10* mRNA/LNPs were labeled with PKH67 (green), and nuclei were stained with DAPI (blue). Scale bars, 50 μm. G) Quantification of intracellular Cy5.5 mean fluorescence intensity. H) Pearson's correlation coefficient analysis of Cy5.5 and PKH67 fluorescence signals. Data are presented as mean ± SD. Statistical significance was determined using one-way ANOVA with Tukey's post-hoc test. *ns*, not significant; ***p < 0.001, ****p < 0.0001.

To systematically evaluate the individual and combined contributions of T peptides and mRNA cargo, a series of nanovesicle-based delivery systems were engineered (Figure 2A). First, S-MNVs and T-MNVs were generated by conjugating MSC-NVs with either scrambled (S) or T peptides, followed by co-extrusion with empty LNPs. Magnetic functionality was achieved by attaching the IONP-Ab to the resulting NV/LNP hybrids (S-MNVs and T-MNVs). To assess mRNA delivery without translation, a non-coding mRNA (*NC* mRNA) was prepared by synthesizing *IL-10* mRNA without a 5′ cap, rendering it non-translatable while retaining its delivery. The *NC* mRNA and *IL-10* mRNA were individually encapsulated into LNPs and co-extruded with T-MNVs to produce *mNC*@T-MNVs and *m10*@T-MNVs, respectively. These were magnetically functionalized to generate *mNC*@T-MNVs and *m10*@T-MNVs, which were the final dual-target magnetic nanocarriers. TEM analysis revealed that all four formulations—S-MNVs, T-MNVs, *mNC*@T-MNVs, and *m10*@T-MNVs—exhibited a spherical morphology with electron-dense cores, indicating successful magnetic nanoparticle integration (Figure 2B). NTA showed similar size distributions across groups, with mean diameters of approximately 110-125 nm (Figure 2C, D). The stepwise modification of the T peptide, *mNC*@T-MNVs, and *m10*@T-MNVs was further validated by zeta potential measurements, demonstrating a gradual change in surface charge at each modification step (Figure 2E). H9C2 cardiomyocytes were treated with nanovesicles constructed by co-extruding Cy5.5-labeled MSC-NVs and PKH67-labeled LNPs to validate the successful synthesis and cardiomyocyte-targeting ability of the hybrid *m10*@T-MNVs. Confocal imaging demonstrated intracellular overlap of Cy5.5 (red) and PKH67 (green) signals in cells treated with T-MNVs-based formulations (Figure 2F). Consistently, T-peptide-modified nanovesicles showed higher Cy5.5 signal intensity than S-MNV, indicating enhanced uptake efficiency conferred by T-peptide modification (Figure 2G). Pearson's correlation coefficient analysis demonstrated comparable colocalization between Cy5.5 and PKH67 signals across groups, indicating that the association between the nanovesicle membrane and the mRNA-containing component of the hybrid nanovesicles was maintained despite differences in uptake levels (Figure 2H). These results validate the structural integrity of the hybrid nanovesicles and the role of T-peptide in facilitating cellular internalization. To elucidate the endocytic pathways involved in the cellular uptake of *m10*@T-MNVs, cells were preincubated with a panel of endocytosis inhibitors prior to treatment with *m10*@T-MNVs. Inhibition of uptake was observed upon pretreatment with wortmannin (50 nM), and 5-(N-ethyl-isopropyl) amiloride (EIPA, 100 μM), indicating reduced internalization of *m10*@T-MNVs (Figure S6A). Quantitative analysis performed at 4 h and 8 h after nanovesicle exposure revealed time-dependent suppression of cellular uptake in the presence of these inhibitors, suggesting that *m10*@T-MNVs are internalized through multiple endocytic pathways, including phagocytosis and macropinocytosis (Figure S6B). To investigate the intracellular trafficking of m10@T-MNVs following cellular uptake, their colocalization with lysosomes was analyzed at different time points. *m10*@T-MNVs showed substantial lysosomal colocalization at 4 h, indicating efficient cellular uptake, whereas the reduced colocalization observed at 8 h is consistent with subsequent intracellular trafficking following internalization (Figure S6C, D).

Before conducting *in vivo* safety studies, we evaluated the *in vitro* cytotoxicity of *m10*@T-MNVs across a range of concentrations (0, 0.5, 1.0, 1.5, and 2.0 µg mL⁻¹). Cell viability and cytotoxicity analyses showed no significant dose-dependent effects of *m10*@T-MNVs across the tested concentration range (Figure S7A, B). These findings indicate that *m10*@T-MNVs exhibit low cytotoxicity and maintains cell viability across the tested concentration range. The mean diameter of *m10*@T-MNVs under different storage conditions (4 °C and -80 °C) and at different time points (Day 0, 7, and 14) was determined to evaluate the stability of *m10*@T-MNVs, showing stability over time (Figure S7C, D). Hemolysis assays were performed to assess biocompatibility and revealed that *m10*@ T-MNVs exhibited minimal hemolytic activity relative to the positive control (Figure S7E).

### Injury-responsive *m10*@T-MNVs targeting and apoptosis attenuation

**Figure 3:**
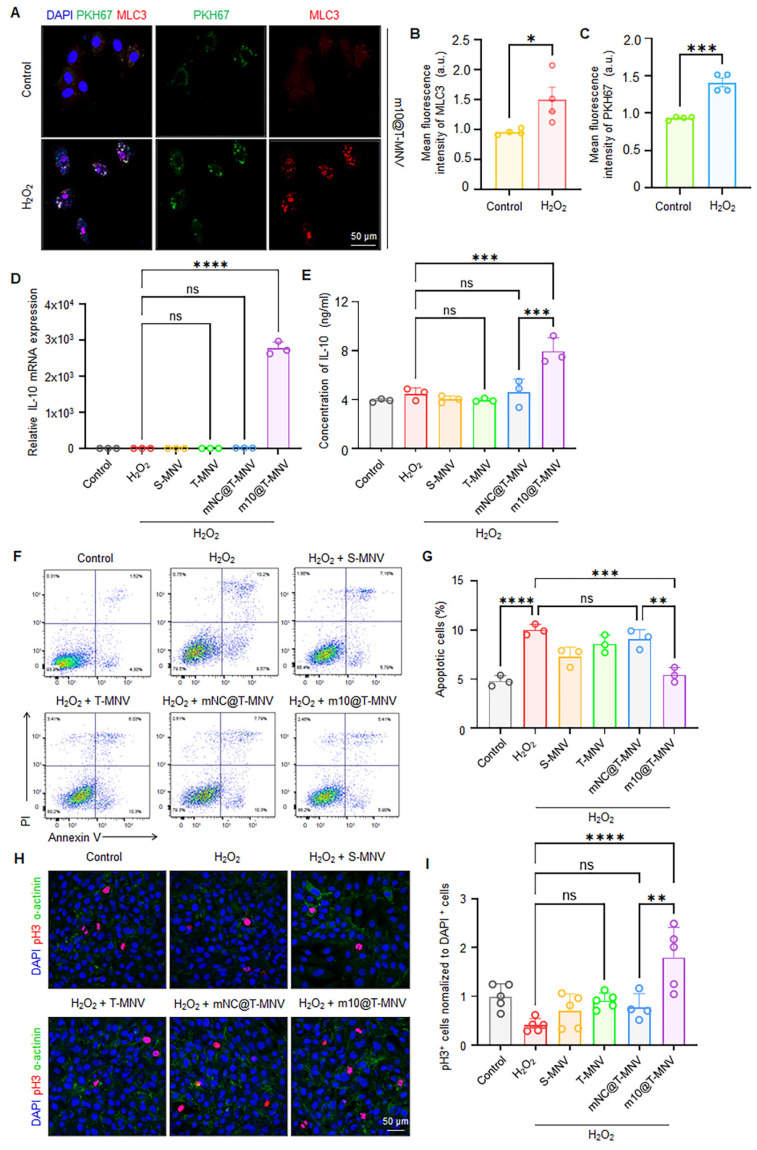
** Injury-responsive targeting and anti-apoptotic effects of m10@T-MNVs.** A) Representative confocal images showing the intracellular distribution of *m10*@T-MNVs (PKH67, green) and MLC3 (red) in H9C2 cardiomyocytes, with or without H₂O₂ treatment. Nuclei were stained with DAPI (blue). Scale bars, 50 μm. B-C) Quantification of fluorescence intensity for MLC3 (B) and PKH67 (C) in cells exposed to H₂O₂ (*n* = 4). D) Relative *IL-10* mRNA expression in cardiomyocytes treated with various nanovesicle formulations, as determined by qRT-PCR (*n* = 3). E) ELISA quantification of IL-10 protein levels in culture supernatants (*n* = 3). F) Representative flow cytometry plots of Annexin V/PI-stained cardiomyocytes after different treatments under oxidative stress. G) Quantification of Annexin V⁺/PI⁺ cardiomyocytes, indicative of late apoptotic or necrotic cells, following treatment with each formulation (*n* = 3). H) Immunofluorescence images of pH3 (red) and sarcomeric α-actinin (green) in H9C2 cells, with DAPI nuclear counterstaining (blue). Scale bars, 50 μm. I) Quantification of pH3⁺ proliferating cardiomyocytes under each condition (*n* = 4). Statistical analysis was performed using two-tailed Student's *t*-test or one-way ANOVA followed by Tukey's post hoc test. Data are presented as mean ± SD. *ns*, not significant; *p < 0.05, **p < 0.01, ***p < 0.001, ****p < 0.0001.

To assess the injury-responsive targeting behavior of *m10*@T-MNVs, cardiomyocytes were exposed to H_2_O_2_ to induce cellular injury. Immunofluorescence imaging demonstrated an increase in MLC3 expression in injured cardiomyocytes, accompanied by enhanced accumulation of PKH67-labeled *m10*@T-MNVs, indicating preferential association of the nanoparticles with injured cells (Figure 3A-C). IL-10 has been reported to exert anti-apoptotic effects in cardiomyocytes and other cell types, supporting its role in cytoprotection under stress conditions 30-32. Based on this rationale, injured cardiomyocytes were subsequently incubated with different nanoparticle formulations to examine IL-10 mRNA delivery. Cells treated with *m10*@T-MNVs exhibited increased *IL-10* mRNA expression (Figure 3D) and corresponding IL-10 protein secretion (Figure 3E), confirming functional mRNA delivery. We evaluated the anti-apoptotic effects of *m10*@T-MNVs using Annexin V/PI staining to assess whether the engineered *m10*@T-MNVs further enhanced these properties. Treatment with *m10*@T-MNVs significantly reduced the proportion of Annexin V^+^/PI^+^ cardiomyocytes, indicative of late apoptosis or necrosis, compared with the H₂O₂ group (Figure 3F, G). Additionally, phosphorylated histone H3 (pH3) immunostaining demonstrated a significant increase in pH3-positive cardiomyocytes in the *m10*@T-MNV-treated group, indicating enhanced cellular recovery (Figure 3H, I). Collectively, these results demonstrate that *m10*@T-MNVs preferentially target injured cardiomyocytes, enable functional *IL-10* mRNA delivery, and attenuate apoptosis under oxidative stress conditions.

### *m10*@T-MNVs promote macrophage polarization toward an anti-inflammatory phenotype

**Figure 4:**
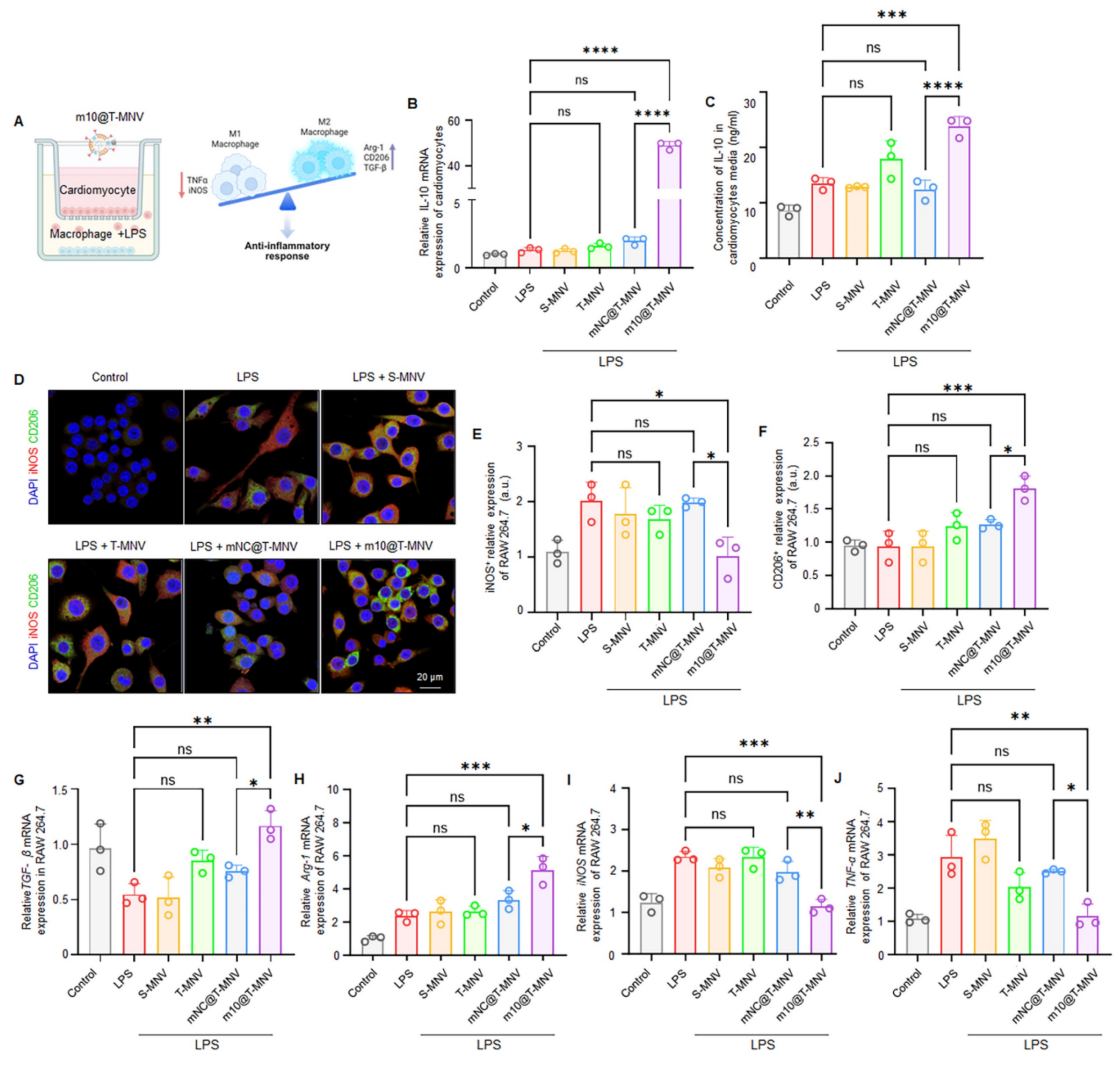
** Macrophage phenotype shifting induced by *m10*@T-MNVs.** A) Schematic illustration of the experimental design: co-culture of LPS-stimulated RAW264.7 macrophages and H9C2 cardiomyocytes followed by treatment with various nanovesicles. B-C) *IL-10* mRNA expression (B) and protein secretion (C) in cardiomyocytes after treatment with indicated formulations, measured by qRT-PCR and ELISA, respectively (*n* = 3). D) Representative immunofluorescence images of macrophages stained for CD206 (green) and iNOS (red), indicating M2 and M1 phenotypes, respectively. Nuclei were counterstained with DAPI (blue). Scale bar, 50 μm. E-F) Quantification of fluorescence intensity for (E) iNOS and (F) CD206 in LPS-stimulated RAW264.7 macrophages treated with the indicated formulations (*n* = 3). G-J) Gene expression analysis of anti-inflammatory markers (G) *TGF-β1*, (H) *Arg1,* and pro-inflammatory markers (I) *iNOS*, (J) *TNF-α* in macrophages across treatment groups. Statistical significance was determined using one-way ANOVA with Tukey's post-hoc test. Data are presented as mean ± SD. *ns*, not significant; *p < 0.05, **p < 0.01, ***p < 0.001, ****p < 0.0001.

We established an *in vitro* co-culture system consisting of H9C2 cardiomyocytes and RAW264.7 macrophages to explore the ability of *m10*@T-MNVs to polarize macrophages *via* cardiomyocyte-targeted IL-10 release. LPS (lipopolysaccharide) was selectively applied to the lower chamber containing RAW264.7 cells to mimic an inflammatory environment, while H9C2 cardiomyocytes in the upper insert remained unstimulated to isolate the paracrine effect of IL-10 following *m10*@T-MNVs treatment (Figure 4A). In this system, *m10*@T-MNVs were efficiently taken up by H9C2 cardiomyocytes, leading to elevated *IL-10* mRNA expression (Figure 4B) and IL-10 protein secretion into the conditioned medium from H9C2 cardiomyocytes (Figure 4C). The conditioned medium was subsequently used to stimulate LPS-treated RAW264.7 macrophages, enabling the assessment of paracrine effects. Immunofluorescence staining revealed increased expression of the M2 macrophage marker CD206 (Figure 4E) and decreased expression of the M1 marker iNOS (Figure 4F), indicating a shift toward an anti-inflammatory phenotype. Consistently, qRT-PCR analysis showed upregulation of anti-inflammatory M2 markers (*TGF-β* and *Arg1*) and downregulation of pro-inflammatory M1 markers (*iNOS* and *TNF-α*) (Figure 4G-J). These findings demonstrate that cardiomyocyte-mediated IL-10 release following *m10*@T-MNVs treatment promoted anti-inflammatory macrophage polarization *in vitro*.

### Targeted delivery of *m10*@T-MNVs into the injured myocardium *in vivo*

**Figure 5:**
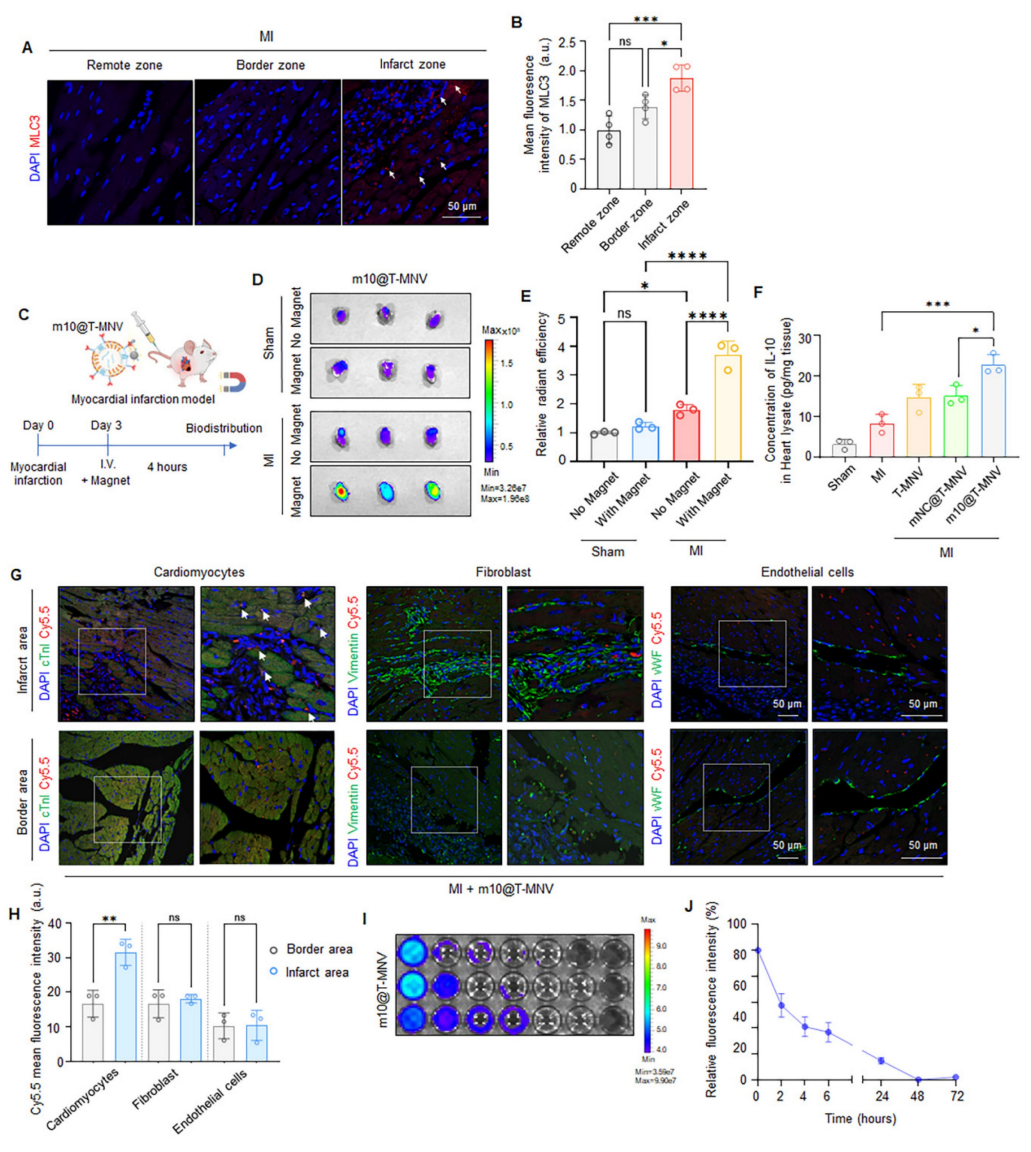
***In vivo* targeting and biodistribution of *m10*@T-MNVs in a mouse model of myocardial infarction.** A) Representative immunofluorescence images showing MLC3 expression in the remote zone, border zone, and infarct zone of infarcted myocardium. Nuclei were stained with DAPI (blue); MLC3, red. Scale bar, 50 μm. B) Quantification of MLC3 fluorescence intensity in the remote zone, border zone, and infarct zone (*n* = 4). C) Schematic illustration of the experimental workflow for *in vivo* biodistribution analysis of m10@T-MNVs in a mouse MI model. D) IVIS^®^ fluorescence images and E) quantification of radiant efficiency in heart tissues 4 h after intravenous injection of Cy5.5-labeled nanoparticles (S-MNVs, T-MNVs, *mNC*@T-MNVs, *m10*@T-MNVs) (*n* = 3). F) ELISA quantification of IL-10 protein levels in mouse hearts (*n* = 4). G) Immunofluorescence images showing uptake of Cy5.5-labeled *m10*@T-MNVs in cardiomyocytes (cTnI), fibroblasts (Vimentin), and endothelial cells (vWF) in the infarct area and border area. Arrowheads indicate Cy5.5⁺ cardiomyocytes. Nuclei were stained with DAPI (blue). Scale bar, 50 μm. H) Quantification of Cy5.5 fluorescence intensity in each cell type in the infarct area and border area (*n* = 3). I) IVIS fluorescence imaging of Cy5.5-labeled *m10*@T-MNVs collected from blood at the indicated time points. J) Time-dependent fluorescence intensity profile of Cy5.5-labeled *m10*@T-MNVs over time (0-72 h) quantified by fluorescence intensity. Statistical analysis was performed using two-tailed Student's *t*-test or one-way ANOVA followed by Tukey's post hoc test. Data are presented as mean ± SD. *ns*, not significant; *p < 0.05, **p < 0.01, ***p < 0.001, and ****p < 0.0001.

MLC3 is a member of the myosin family and is released from the cytoplasm of cardiomyocytes upon severe myocardial injury 14, 33. Accordingly, we examined MLC3 protein profiles in infarcted cardiac tissues. Western blot analysis revealed increased MLC3 immunoreactivity in MI hearts compared with sham controls (Figure S8). To further localize this increase, immunohistochemistry was performed on heart sections from MI mice to confirm that MLC3 was upregulated in the infarcted region (Figure 5A). MLC3 expression was significantly higher in the infarct zone than in the border zone and the remote zone (Figure 5B), supporting its potential as a molecular target for site-specific delivery to injured cardiac tissues.

Next, we intravenously injected the MNPs into sham and MI mice on day 3 post-infarction and assessed their accumulation in the infarcted cardiac tissue to determine whether the *m10*@T-MNVs effectively targeted the injured myocardium. Cy5.5-labeled MNPs were intravenously injected into healthy mice with (MF+ group), or without an external MF (MF- group), applied to the heart to evaluate whether magnetic guidance alone enabled cardiac targeting *in vivo* (Figure S9A). In vivo Imaging System (IVIS) imaging showed a marked increase in cardiac fluorescence in the MF+ group, whereas fluorescence signals in other major organs remained comparable between the groups (Figure S9B-E). Histological and biochemical analyses were performed after MNP administration and magnetic field exposure to evaluate the potential toxicity of the MNPs. Histological and hematological analyses revealed no signs of organ toxicity following the MNPs injection or magnetic exposure (Figure S9F, G). These findings suggested that antibody-conjugated MNPs can be safely and effectively guided to the heart. MNPs were intravenously injected into both sham and MI mice, with or without the application of an external magnetic field, to assess whether *m10*@T-MNVs can facilitate injured cardiac targeting *in vivo* (Figure 5C). Magnetic field application selectively enhanced cardiac accumulation of *m10*@T-MNVs in MI mice, confirming the effectiveness of magnetic guidance for targeted delivery to the infarcted myocardium (Figure 5D, E and Figure S10). Next, Cy5.5-labeled *m10*@T-MNVs were intravenously injected into MI mice, followed by the application of an external magnetic field, to determine whether the *m10*@T-MNVs could specifically target the heart *in vivo.* Biodistribution analysis further confirmed significantly enhanced cardiac accumulation of *m10*@T-MNVs compared with S-MNVs (Figure S11). These findings indicate that T-peptide-mediated targeting contributes to the preferential accumulation of *m10*@T-MNVs in heart tissues. To further validate cardiac-specific delivery at the protein level, IL-10 secretion was quantified using enzyme-linked immunosorbent assay (ELISA) in various organs of MI mice, including the heart, lungs, kidneys, spleen, and liver. *m10*@T-MNVs administration led to a significant increase in IL-10 expression in the infarcted heart compared to MI mice, whereas other organs did not exhibit a comparable IL-10 upregulation relative to their respective controls. (Figure 5F, Figure S12). These findings suggested that *m10*@T-MNV cardiac-targeted delivery effectively induced IL-10 secretion.

To identify the specific cardiac cell types targeted by *m10*@T-MNVs, immunofluorescence staining was performed on heart sections using markers for cardiomyocytes (cardiac troponin I, cTnI), fibroblasts (vimentin), and endothelial cells (von Willebrand factor, vWF), respectively. *m10*@T-MNVs showed preferential association with cardiomyocytes, as evidenced by cTnI⁺ signals, whereas minimal uptake was observed in vimentin⁺ fibroblasts or vWF⁺ endothelial cells (Figure 5G, H). Fluorescence intensity in the blood was monitored over time following intravenous injection into MI mice to evaluate the in vivo circulation and clearance kinetics of *m10*@T-MNVs (Figure 5I). Quantitative analysis revealed a rapid decline within the first 6 h and near-complete clearance after 48 h (Figure 5J). Taken together, the data reveal preferential cardiac accumulation of m10@T-MNVs, particularly within cardiomyocytes, indicating their potential as heart-targeted delivery systems. Moreover, histological and hematological analyses were conducted 4 h after injection to assess the safety of *m10*@T-MNVs as a nanocarrier. Hematoxylin and eosin (H&E) staining showed no significant tissue alterations in the major organs, and blood tests revealed no changes in glutamate oxaloacetate transaminase (GOT), glutamate pyruvate transaminase (GPT), alkaline phosphatase (ALP), or albumin (ALB) levels across the treatment groups (Figure S13). These results indicated that *m10*@T-MNVs were efficiently delivered to the heart without inducing systemic toxicity, supporting their potential as safe therapeutic nanoplatforms. Collectively, these findings demonstrated that *m10*@T-MNVs enabled effective delivery to the injured myocardium *via* magnetic and molecular targeting, with predominant accumulation in cardiomyocytes. This system ensured functional *IL-10* mRNA expression in the injured heart, exhibited favorable *in vivo* safety profile, and showed minimal systemic toxicity, supporting its potential as a safe therapeutic nanoplatform that targets injured cardiomyocytes.

### *m10*@T-MNVs modulate macrophage polarization and inflammatory responses *in vivo*

**Figure 6:**
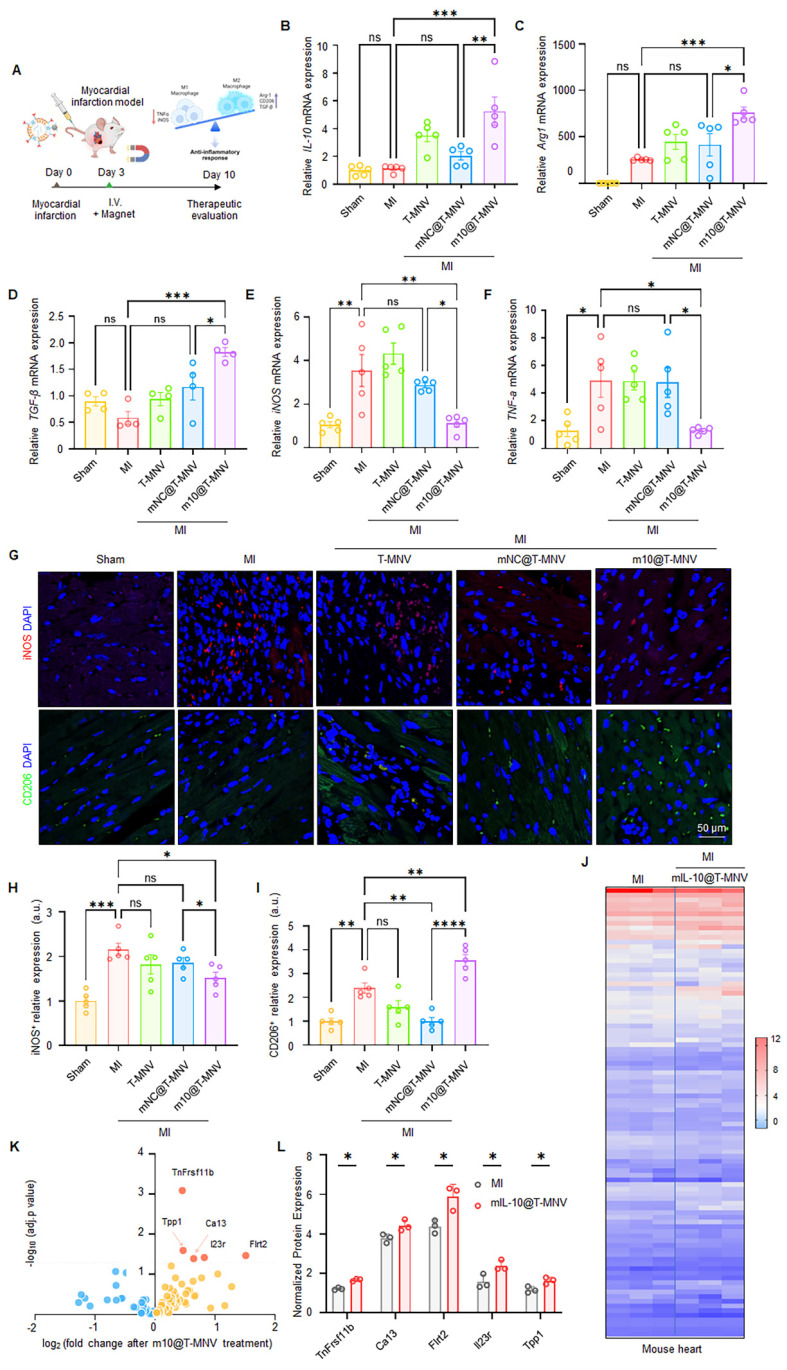
** Macrophage phenotype switching and proteomic remodeling induced by *m10*@T-MNVs in a mouse model of myocardial infarction.** A) Conceptual schematic depicting intravenous administration of *m10*@T-MNVs for *in vivo* therapeutic delivery following MI. B) qRT-PCR analysis of *IL-10* mRNA in infarcted heart tissues (*n* = 5). C-F) qRT-PCR analysis of macrophage polarization markers in infarcted heart tissues, including (C) *Arg1*, (D) *TGF-β*, (E) *iNOS*, and (F) *TNF-α* (*n* = 5). G) Immunofluorescence staining of infarcted myocardium showing iNOS (red) and CD206 (green) expression in tissue sections from indicated groups. Nuclei were counterstained with DAPI (blue). Scale bar, 50 μm. H-I) Quantification of (H) iNOS and (I) CD206 fluorescence intensity in infarcted hearts (*n* = 5). J-L) Protein expression analysis of infarcted cardiac tissues after treatment with *m10*@T-MNVs (*n* = 3). J**)** Heatmap displaying hierarchical clustering of inflammation-associated proteins significantly altered by *m10*@T-MNV treatment. K**)** Volcano plot showing differentially expressed proteins in hearts treated with *m10*@T-MNVs compared with untreated MI controls. L**)** Normalized protein expression levels of significantly altered proteins identified by targeted proteomic analysis. Statistical significance was determined using two-tailed Student's *t*-test or one-way ANOVA with Tukey's post-hoc test. Data are presented as mean ± SD. *ns*, not significant; *p < 0.05, **p < 0.01, ***p < 0.001, and ****p < 0.0001.

We administered various nanoparticle formulations to mice with MI and assessed the expression of inflammatory and anti-inflammatory genes to evaluate the immunomodulatory effects of *m10*@T-MNVs following MI (Figure 6A). Intravenous injection of *m10*@T-MNVs elevated *IL-10* mRNA levels in heart tissues (Figure 6B). The qRT-PCR results revealed the upregulation of anti-inflammatory M2 markers (*TGF-β* and *Arg1*) and downregulation of pro-inflammatory M1 markers (*iNOS* and *TNF-α*), confirming a *m10*@T-MNVs-mediated anti-inflammatory response (Figure 6C-F). Immunofluorescence staining confirmed these findings, showing reduced iNOS (M1 marker) and increased CD206 (M2 marker) expression in *m10*@T-MNVs-treated hearts, supporting effective macrophage repolarization toward an anti-inflammatory phenotype (Figure 6G-I). This response influenced cardiac macrophage polarization toward an anti-inflammatory phenotype, contributing to the suppression of inflammation in myocardial tissue. To further elucidate the mechanistic basis of *m10*@T-MNVs-induced cardioprotection, proteomic profiling of infarcted cardiac tissue was performed (Figure 6J). Notably, *m10*@T-MNVs treatment upregulated several protective proteins such as TNFRSF11B (Tumor necrosis factor receptor superfamily member 11B), TPP1 (Tripeptidyl peptidase 1), CA13 (Carbonic anhydrase 13), IL23R (Interleukin-23 receptor), and FLRT2 (Fibronectin leucine rich transmembrane protein 2), which are associated with oxidative stress mitigation, immune modulation, cardiac tissue repair, enhanced cell survival, and anti-inflammatory responses (Figure 6K, L). These findings suggest that beyond suppressing inflammatory cascades, *m10*@T-MNVs facilitate cardioprotective remodeling and microenvironmental stabilization post-MI. Collectively, these findings indicated that *m10*@T-MNVs contribute to anti-inflammatory modulation and may support the molecular pathways associated with myocardial repair following infarction.

### *m10*@T-MNVs treatment improves cardiac function and reduces infarct size

**Figure 7:**
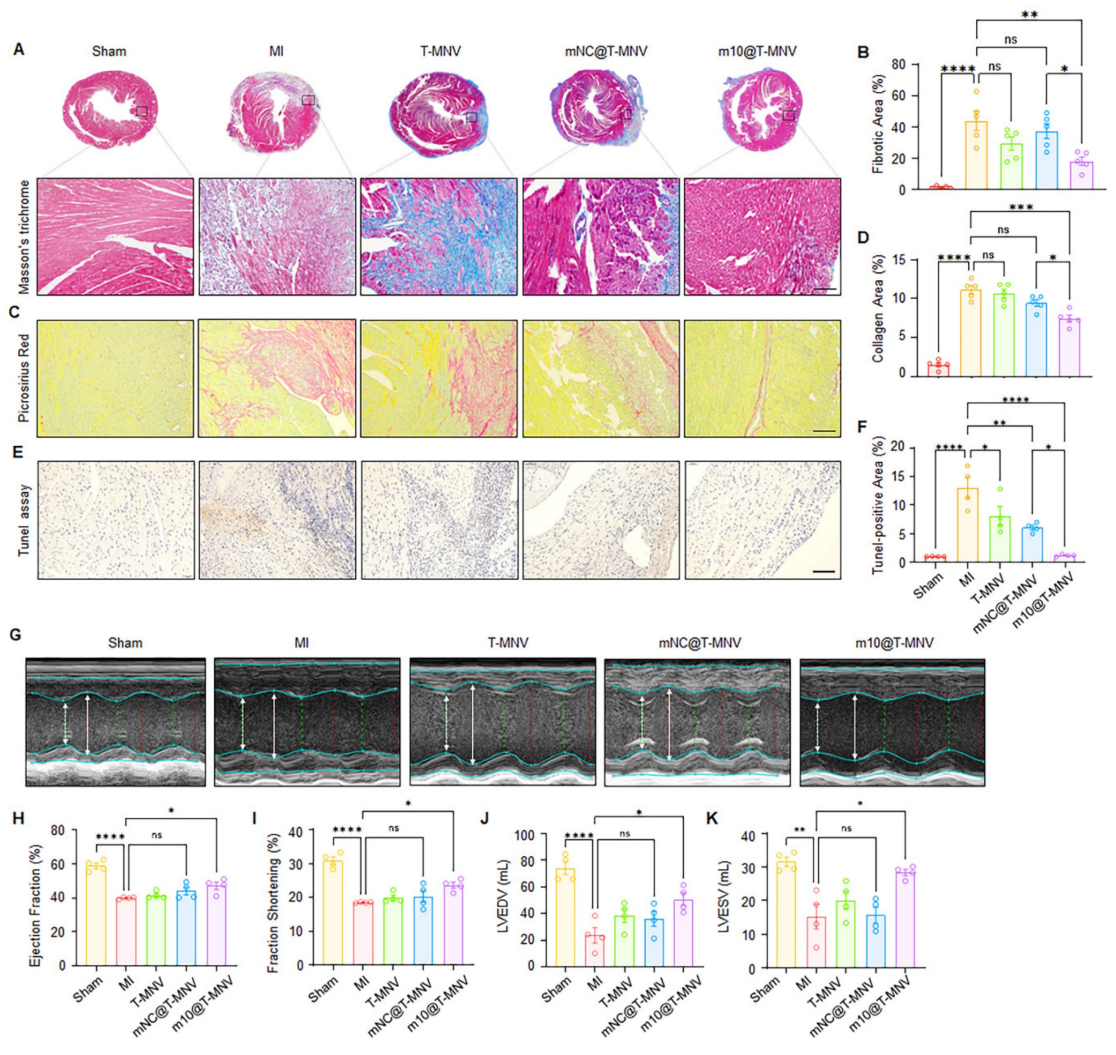
** Effects of *m10*@T-MNVs in a mouse model of myocardial infarction.** A) Representative histological images of hearts stained with Masson's trichrome and B) quantification of the fibrotic area. The enlarged image is a magnified image of the black dashed line-boxed area. Scale bars, 25 μm (*n* = 5). C) Representative histological images of Sirius Red and D) Quantification of collagen area (%). Scale bars, 25 μm (*n* = 5). E) Representative photomicrographs of terminal deoxynucleotidyl transferase dUTP nick-end labeling (TUNEL) staining of myocardial sections. Scale bars, 25 μm. F) Quantification of TUNEL-positive area (%) (*n* = 4). G) Representative M-mode echocardiography images of hearts from different treatment groups. H-K) Quantification of cardiac function parameters, including (H) ejection fraction (%), (I) fractional shortening (%), (J) left ventricular end-diastolic volume (LVEDV, mL), and (K) left ventricular end-systolic volume (LVESV, mL) (*n* = 4). Statistical significance was determined using one-way ANOVA with Tukey's post-hoc test. Data are presented as mean ± SD. *ns*, not significant; LVEDV, left ventricular end-diastolic volume; LVESV, left ventricular end-systolic volume; *p < 0.05, **p < 0.01, ***p < 0.001, and ****p < 0.0001.

Next, we evaluated whether treatment with *m10*@T-MNVs improved post-MI cardiac repair and functional recovery. In this study, MI mice were systemically administered *m10*@T-MNVs, and a series of histological and functional assessments were conducted to evaluate therapeutic outcomes. Masson's trichrome staining revealed a reduction in the infarct area in the *m10*@T-MNVs-injected group compared with that in the *mNC*@T-MNV-injected group, indicating suppression of infarct-induced scar formation (Figure 7A, B). Consistent with the improved scar area, Sirius Red staining revealed significantly reduced collagen deposition in the infarct region following *m10*@T-MNVs treatment (Figure 7C, D). The terminal deoxynucleotidyl transferase dUTP nick-end labelling (TUNEL) assay demonstrated a decreased number of apoptotic cells in the *m10*@T-MNVs group, suggesting protection against ischemia-induced cardiomyocyte death (Figure 7E, F). Furthermore, echocardiographic assessment revealed significant improvements in the ejection fraction and fractional shortening, along with reductions in LVEDV (Left Ventricular End-Diastolic Volume) and LVESV (Left Ventricular End-Systolic Volume) in the *m10*@T-MNVs-treated mice, indicating improved systolic function and ventricular remodeling (Figure 7G-K). Collectively, these data demonstrated that *m10*@T-MNVs enhance cardiac repair by mitigating fibrosis and apoptosis, thereby promoting functional recovery after MI and highlighting their therapeutic utility as targeted mRNA delivery platforms for cardiovascular diseases.

### *m10*@T-MNVs attenuate angiotensin II-induced myocardial fibrosis and improve cardiac function

**Figure 8:**
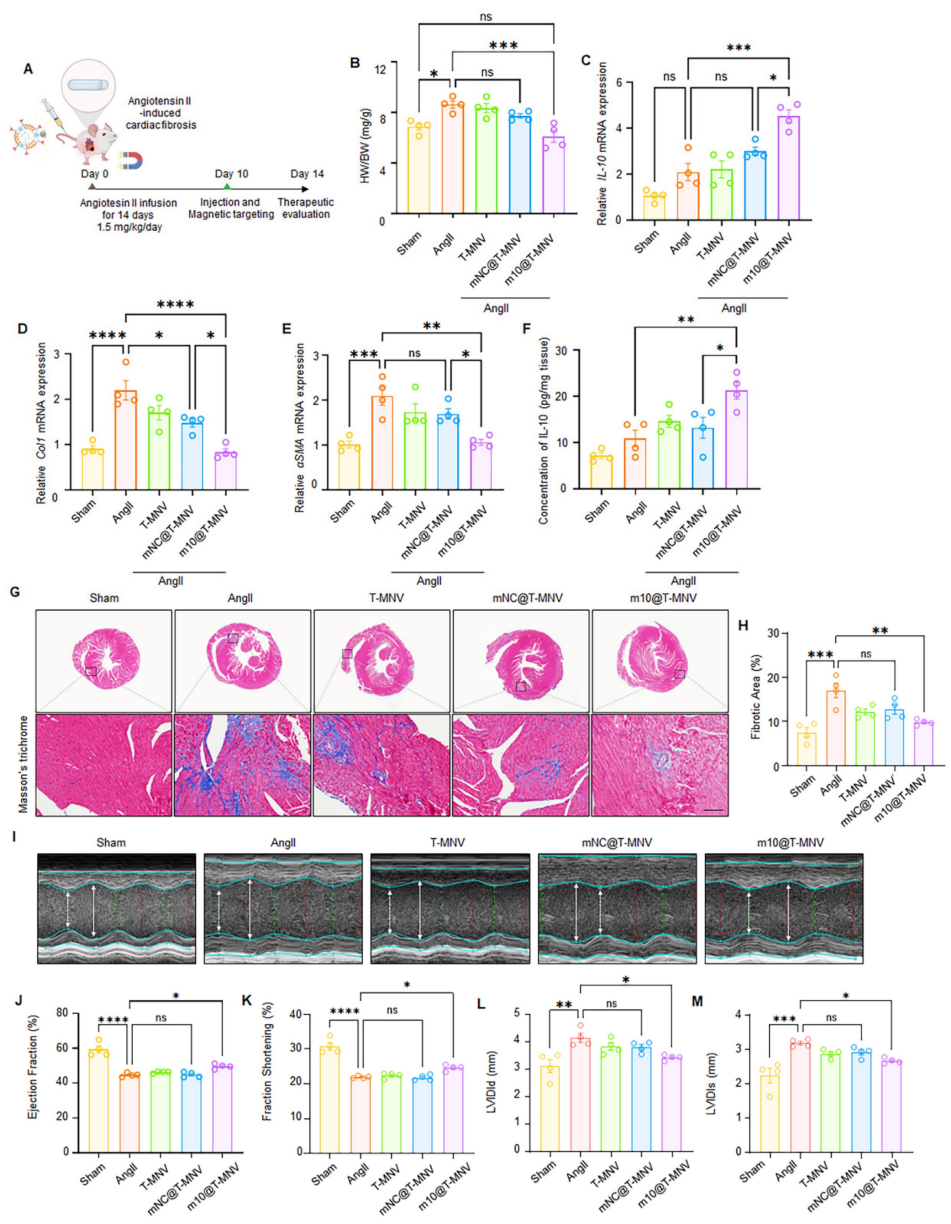
** Evaluation of *m10*@T-MNVs in an angiotensin II-induced cardiac fibrosis model.** A) Schematic illustration of *m10*@T-MNVs *via* intravenous injection for the treatment of myocardial fibrosis injury. B) Heart-weight-to-body weight (HW/BW) ratio. C) qRT-PCR analysis of *IL-10* mRNA in heart tissues (*n* = 4). D-E) qRT-PCR analysis of fibrosis markers in infarcted heart tissues, including (D) *collagen I*, and (E) *α-SMA* (*n* = 4). (F) ELISA quantification of IL-10 protein levels in mouse hearts (*n* = 4). G) Representative histological image of hearts stained with Masson's trichrome and (H) quantification of the fibrotic area (*n* = 4). Scale bars, 25 μm. I) Representative M-mode echocardiography images of hearts from different treatment groups (*n* = 4). J-M) Quantification of cardiac function parameters, including (J) ejection fraction (%), (K) fractional shortening (%), (L) left ventricular internal diameter at end-diastole (LVIDd), and (M) left ventricular internal diameter at end-systole (LVIDs). Statistical significance was determined using one-way ANOVA with Tukey's post-hoc test. Data are presented as mean ± SD. *ns*, not significant; *p < 0.05, **p < 0.01, ***p < 0.001, and ****p < 0.0001.

Chronic elevation of angiotensin II (Ang II) levels is known to trigger myocardial fibrosis 34, 35. To evaluate the therapeutic efficacy of *m10*@T-MNVs in a fibrotic setting, an Ang II-induced cardiac fibrosis model was employed. Mice were continuously infused with Ang II (2 mg·kg⁻¹·day⁻¹) for 2 weeks using osmotic pumps, followed by intravenous administration of the indicated nanovesicle formulations (Figure 8A). Ang II infusion increased the heart-weight-to-body-weight (HW/BW) ratio compared with the sham group, whereas mice treated with *m10*@T-MNVs showed a reduced HW/BW ratio relative to the Ang II group (Figure 8B). Intravenous injection of *m10*@T-MNVs resulted in higher *IL-10* mRNA levels in heart tissues compared with other treatment groups (Figure 8C). The qRT-PCR results revealed upregulation of fibrosis-associated markers (*collagen I* and *α-SMA*) in Ang II-infused hearts, whereas *m10*@T-MNVs treatment suppressed their expression, indicating attenuation of myocardial fibrosis (Figure 8D, E). ELISA analysis demonstrated increased IL-10 protein levels in heart tissues from *m10*@T-MNVs-treated mice compared with the Ang II group (Figure 8F). Histological evaluation using Masson's trichrome staining revealed extensive fibrotic areas in Ang II-infused hearts, whereas reduced fibrotic deposition was observed in the *m10*@T-MNVs group (Figure 8G, H). Furthermore, echocardiographic assessment revealed significant improvements in the ejection fraction and fractional shortening, along with reductions in left ventricular internal diameter at end-diastole (LVIDd), and left ventricular internal diameter at end-systole (LVIDs) in the *m10*@T-MNVs-treated mice, indicating improved systolic function and ventricular remodeling (Figure 8I-M). Collectively, these data demonstrated that *m10*@T-MNVs enhance cardiac repair by mitigating fibrosis, thereby promoting functional recovery after myocardial fibrosis.

## Conclusions

Targeted delivery of mRNA therapeutics remains a major challenge in the treatment of cardiac diseases, particularly because of the complex cellular composition and limited permeability of infarcted myocardial tissues. These pathological features hinder the efficient accumulation and cellular uptake of mRNA nanocarriers at the injury site. IL-10 is a central anti-inflammatory cytokine that modulates immune responses and promotes a prototypical M2-like macrophage phenotype, thereby supporting tissue repair after MI. Accordingly, IL-10 administration is considered a potential treatment for patients after MI. In this study, we engineered a hybrid nanocarrier, *m10*@T-MNVs, that integrates magnetic guidance with cardiomyocyte-specific targeting to enable selective delivery of *IL-10* mRNA to the injured heart. This platform incorporates three synergistic modules: (1) MSC-NVs to enhance biocompatibility and immune evasion. (2) magnetic nanoparticles to promote externally guided cardiac accumulation, and (3) MLC3 antibodies and T peptides to achieve injured cardiomyocyte-specific targeting. The integration of these features enabled precise mRNA delivery into injured cardiomyocytes, leading to enhanced IL-10 expression and improved post-infarction cardiac remodeling. Consequently, *m10*@T-MNVs induced localized immunomodulation within the infarct zone and significantly improved cardiac outcomes in a murine MI model. Further mechanistic dissection using single-cell RNA seqeuncing and validation in larger-animal models will be important for future translation.

In summary, this study presents a rational design strategy for mRNA nanocarriers capable of achieving spatially and cellularly controlled delivery to diseased tissues. Beyond cardiac repair, the adaptability of this platform to diverse therapeutic cargos and target organs underscores its broader translational potential. As mRNA therapeutics continue to expand beyond vaccines, precision delivery systems may provide a versatile framework for treating cardiovascular and other inflammation-associated diseases.

## Supplementary Material

Supplementary figures and tables.

## Abbreviations

MI: myocardial infarction
mRNA: messenger RNA
IL-10: interleukin-10
IONP: iron oxide nanoparticle
IONP-Ab: antibody-conjugated IONPs
MLC3: myosin light chain 3
MSC: mesenchymal stem cell
MSC-NV: mesenchymal stem cell-derived nanovesicle
MNV: magnetic nanovesicle
T peptides: cardiac-targeting peptides
S-MNV: scrambled peptide-modified magnetic nanovesicle
T-MNV: T peptide-modified magnetic nanovesicle
*mNC*@T-MNV: negative control mRNA-loaded T-MNV
*m10*@T-MNV: IL-10 mRNA-loaded T-MNV
Ang II: angiotensin II
