# Supplementary material for: Injured cardiac targeting magnetic nanovesicles for mRNA treatment of myocardial infarction
Source: Theranostics. 2026 Jan 21;16(8):4090–112. doi: 10.7150/thno.124754 (PMC12905742; doi:10.7150/thno.124754)
Supplement: Supplementary file 1 — Supplementary figures and tables. [file thnov16p4090s1.pdf]

Supporting Information

Injured cardiac targeting magnetic nanovesicles for mRNA treatment of myocardial infarction

Dasom Mun[1], Ji-Young Kang[1], Malgeum Park[1], Gyeongseo Yoo[1], Jaewoong Lee[1], Nuri Yun [2*], Boyoung Joung[1*]

[1] Division of Cardiology, Yonsei University College of Medicine, Seoul 03722, Republic of Korea

[2] GNTPharma Science and Technology Center for Health, GNTPharma Incheon 21983, Republic of Korea

[*]Corresponding authors: Nuri Yun, E-mail: yunnuri@hanmail.net; Boyoung Joung, E-mail: cby6908@yuhs.ac

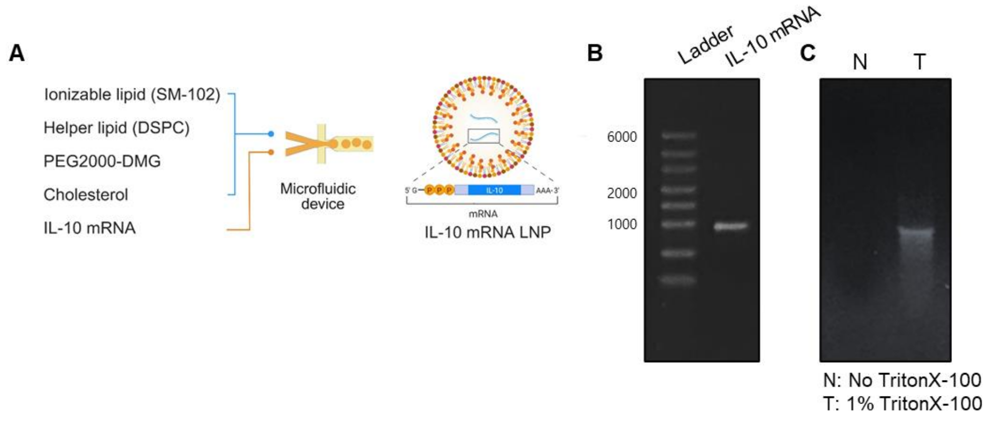

Figure S1. Preparation and characterization of *IL-10* mRNA-loaded lipid nanoparticles. A) Schematic representation of the formulation of *IL-10* mRNA LNPs using a microfluidic device. Lipid components including ionizable lipid (SM-102), helper lipid (DSPC), cholesterol, and PEG-lipid (PEG2000-DMG) were combined with *IL-10* mRNA to produce lipid nanoparticles via rapid mixing. B) Agarose gel electrophoresis confirming the integrity of *IL-10* mRNA. C) Gel retardation assay demonstrating encapsulation of *IL-10* mRNA within LNPs. Lane N: *IL-10* mRNA LNPs treated without Triton X-100 ; Lane T: *IL-10* mRNA LNPs treated with Triton X-100.

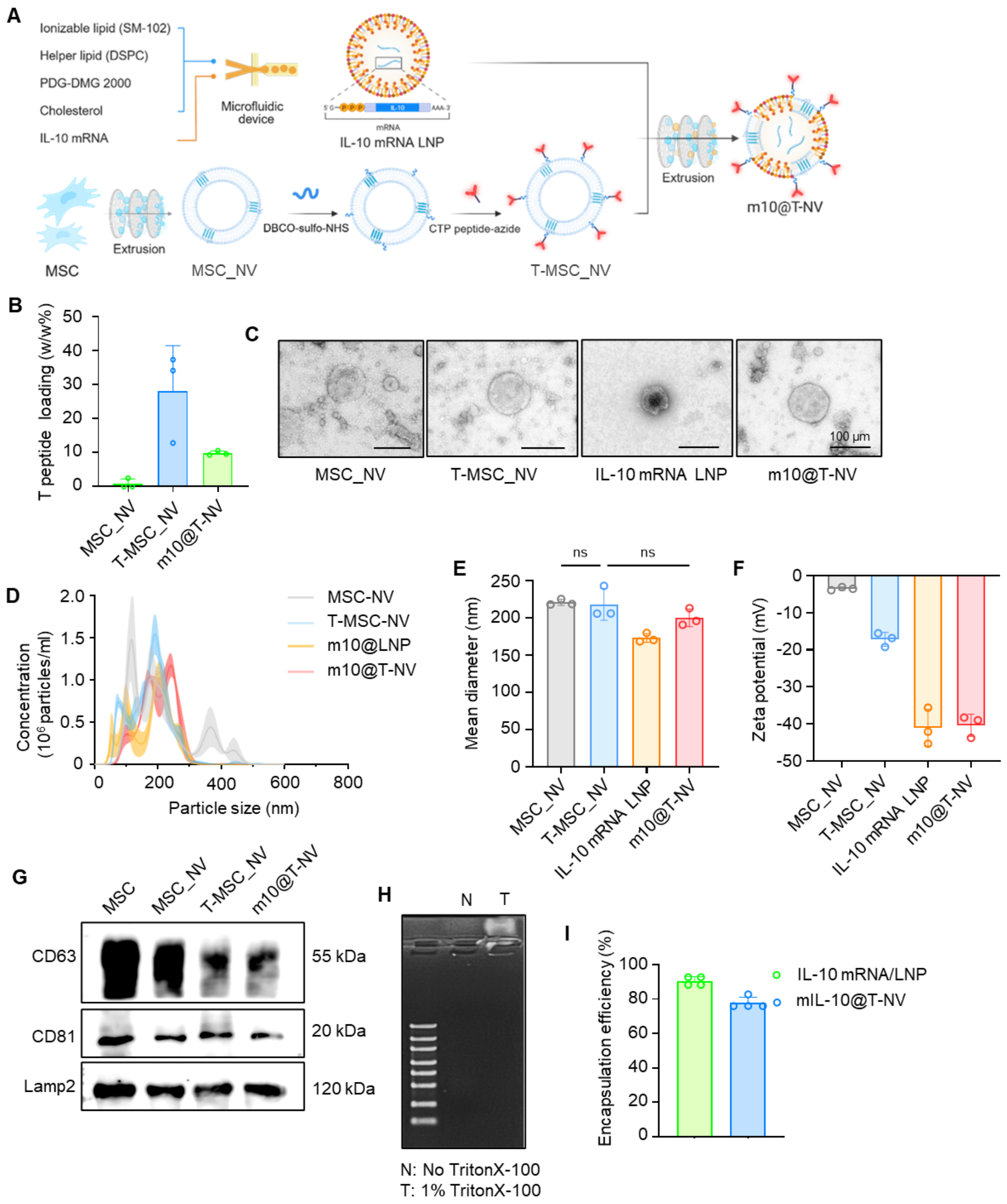

Figure S2. Characterization of hybrid IL-10 mRNA-loaded and cardiac targeting peptide-modified nanovesicles. A) Schematic showing the stepwise assembly of *m10*@T-NVs by extruding MSC-derived nanovesicles (MSC_NVs), conjugating cardiac targeting peptides (T peptides) via DBCO-sulfo-NHS and azide click chemistry to yield T-MSC_NVs, and co-extrusion with IL-10 mRNA/LNPs. B) Quantification of T peptide loading efficiency (w/w%) on NVs, MSC_NVs, and *m10*@T-NVs (*n* = 3). C) Transmission electron microscopy images of MSC_NVs, T-MSC_NVs, IL-10 mRNA LNPs, and *m10*@T-NVs. Scale bars: 100 nm. D) Nanoparticle tracking analysis (NTA) showing size distribution profiles of each nanoparticle formulation. E) Mean hydrodynamic diameter of nanoparticles determined by dynamic light scattering. F) Zeta potential measurements of each formulation indicating surface charge

profiles. G) Western blot analysis of MSC cell-derived markers (CD63, CD81, LAMP2) across different formulations. H) Gel retardation assay to evaluate *IL-10* mRNA encapsulation in *m10*@T-NVs with and without Triton X-100 treatment. I) Encapsulation efficiency of *IL-10* mRNA in LNPs alone and in hybrid *m10*@T-NVs. Statistical significance was determined using one-way ANOVA with Tukey's post-hoc test. Data are presented as mean ± SD. *ns*, not significant.

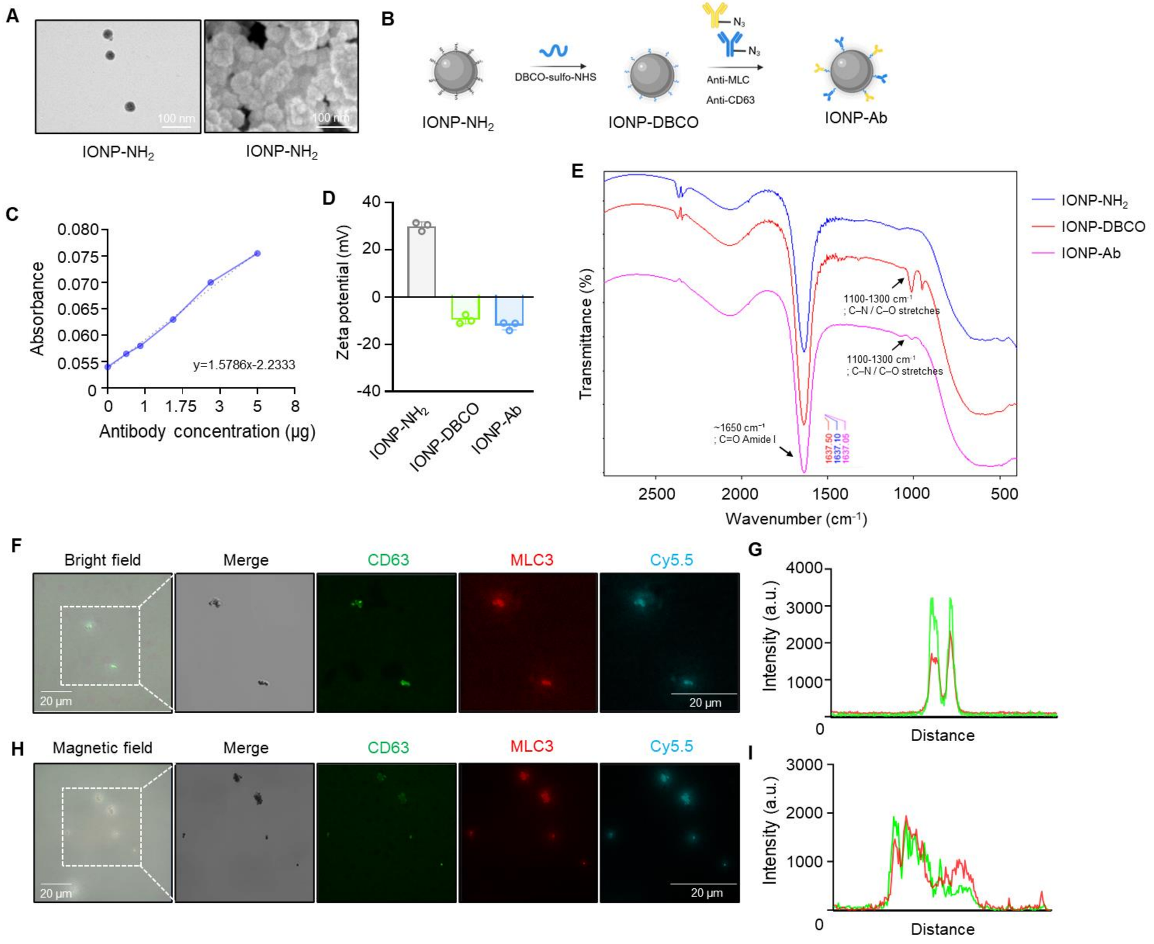

Figure S3. Synthesis and characterization of antibody-conjugated magnetic nanovesicles and their *in vitro* cardiac targeting under magnetic guidance. A) Transmission electron microscopy and scanning electron microscopy analyses of IONP-NH_2. Scale bars: 100 nm. B) Schematic representation of surface modification steps: functionalization of IONP-NH_2 with DBCO-sulfo-NHS and subsequent click conjugation with azide-modified antibodies (anti-MLC3 or anti-CD63) to form IONP-Abs. C) Standard curve plotting absorbance against antibody concentration for quantification of conjugated antibodies. D) Zeta potential measurements of IONP-NH_2, IONP-DBCO, and IONP-Abs. E) Fourier-transform infrared (FTIR) spectra of IONP-NH_2, IONP-DBCO, and IONP-Abs. F) Bright field and fluorescence images of Cy5.5-labeled IONP-Abs (blue) and immunostained for CD63 (green), MLC3 (red). Scale bars: 20 μm. G) Fluorescence intensity profiles of CD63, and MLC3 signals. H) Bright field and fluorescence images of IONP-Ab labeled with Cy5.5 (blue) and immunostained for CD63 (green), MLC3 (red) under external magnetic field application. I) Fluorescence intensity profiles under magnetic guidance. Scale bars: 20 μm.

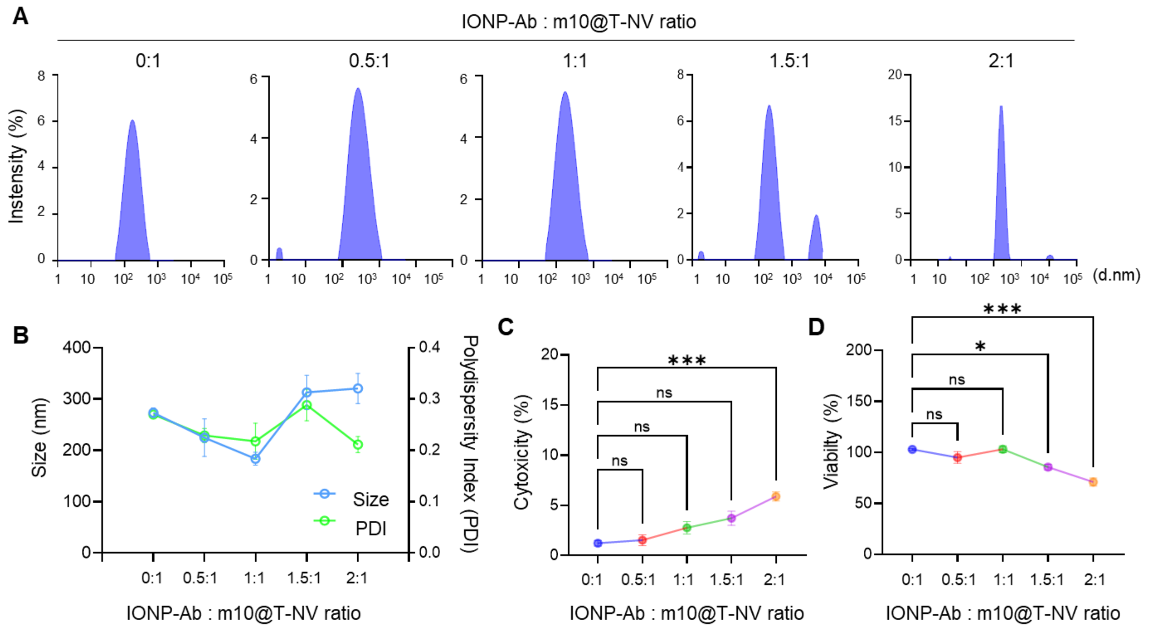

Figure S4. IONP-Ab:*m10*@T-NV ratio–dependent screening of hybrid nanovesicle formation. A) Dynamic light scattering (DLS) measurements of hybrid vesicles generated at increasing IONP-Ab:*m10*@T-NV ratios. B) Quantitative comparison of hydrodynamic diameter and polydispersity index (PDI). C) Cytotoxicity analysis of hybrid nanovesicles prepared by combining antibody-functionalized magnetic nanoparticles (IONP-Abs) with *m10*@T-NVs at different IONP-Ab:*m10*@T-NV ratios (0:1–2:1). D) Cell viability assessment of hybrid nanovesicles prepared at the indicated IONP-Ab:*m10*@T-NV ratios. Statistical analyses were performed using one-way ANOVA with Tukey's post-hoc test. *$p < 0.05$, **$p < 0.01$, ***$p < 0.001$; ns, not significant.

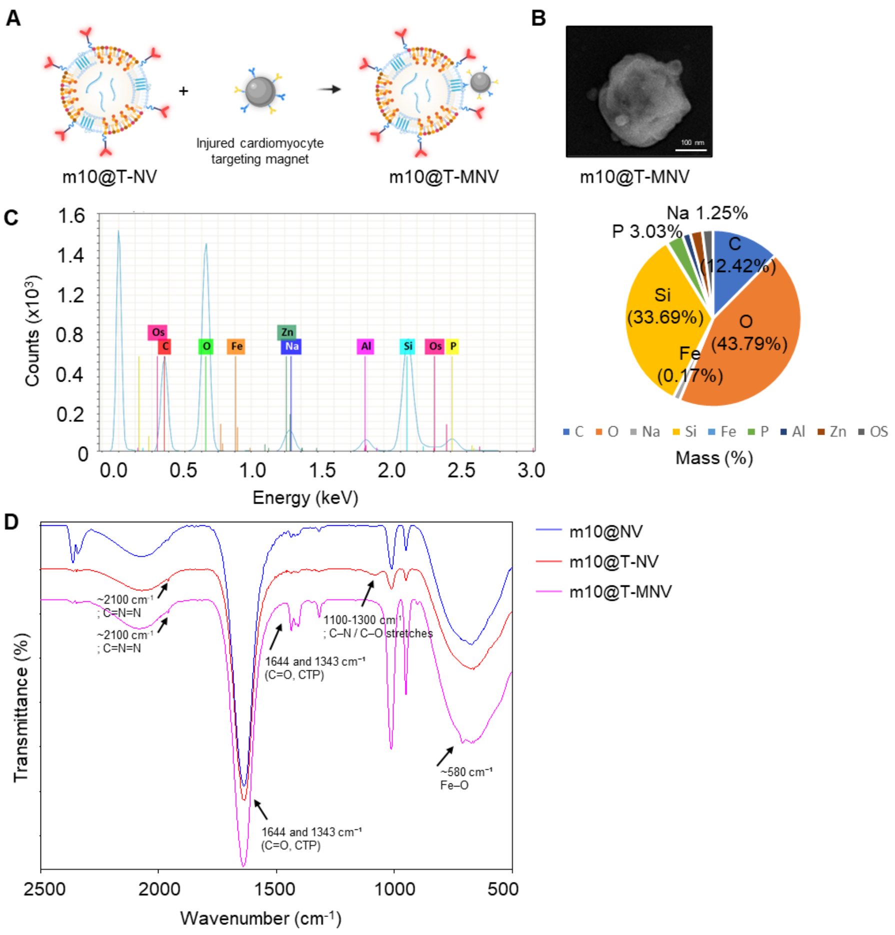

Figure S5. Schematic construction and surface characterization of *m10*@T-MNVs. A) Schematic illustration of *m10*@T-MNVs formation by hybridizing *m10*@T-NVs with magnetic nanoparticles (IONP-Abs). B) Representative scanning electron microscopy (SEM) image of *m10*@T-MNVs. Scale bar: 100 nm. C) Energy-dispersive X-ray spectroscopy (EDS) spectrum and corresponding elemental composition pie chart showing atomic content of *m10*@T-MNVs. D) Fourier-transform infrared (FTIR) spectra of *m10*@NVs, *m10*@T-NVs, and *m10*@T-MNVs.

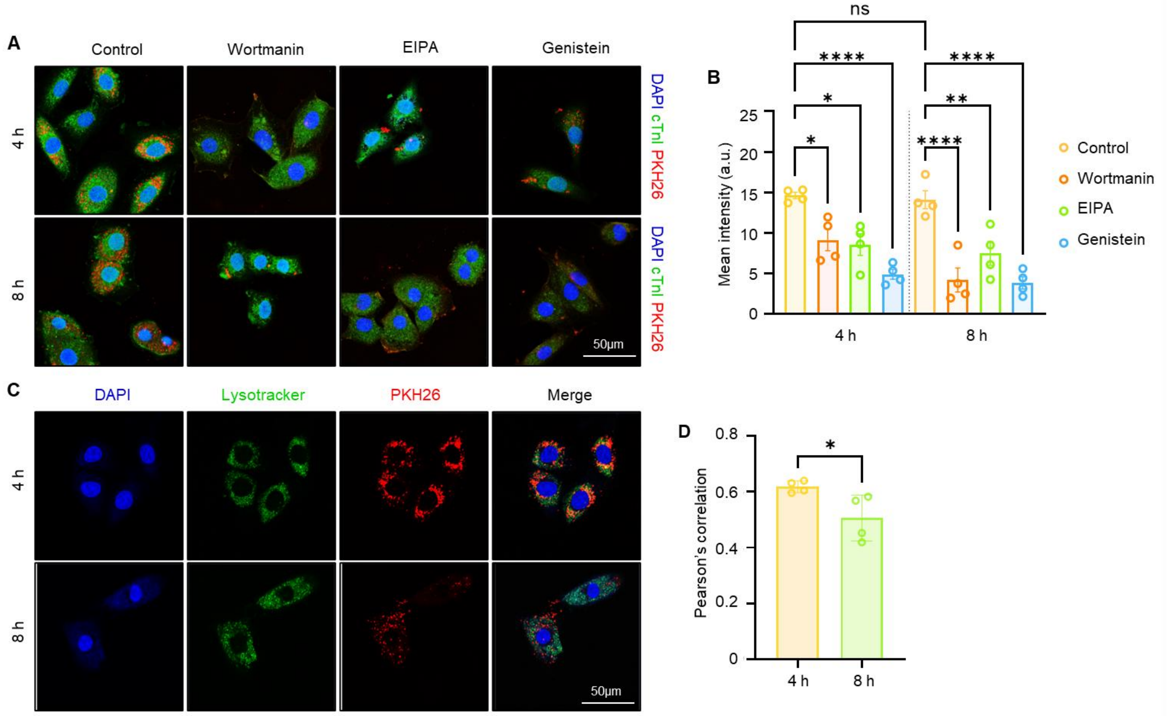

Figure S6. Intercellular trafficking and endosomal escape of *m10*@T-MNVs. A) Immunofluorescence images showing the internalization of PKH26-labeled *m10*@T-MNVs after preincubation with endocytosis inhibitors (wortmannin, 5-(N-ethyl-N-isopropyl) amiloride (EIPA), and genistein). Scale bars, 50 µm. B) Quantification of intracellular uptake of PKH26-labeled *m10*@T-MNVs following treatment with endocytosis inhibitors at 4 h and 8 h. Mean fluorescence intensity of PKH26 was quantified from confocal images for each condition (control, wortmannin, EIPA, and genistein). C) Immunofluorescence images of H9C2 cells incubated with Cy5.5-labeled *m10*@T-MNVs for 4 h and 8 h. Nuclei were stained with Hoechst (blue), and lysosomes were visualized using LysoTracker (green). Scale bars, 50 µm. D) Quantification of the colocalization between *m10*@T-MNVs and lysosomes as an indicator of intracellular trafficking following cellular uptake. Statistical significance was determined using one-way ANOVA with Tukey's post-hoc test. Data are presented as mean ± SD. ns, not significant; *$p < 0.05$, **$p < 0.01$, and ****$p < 0.0001$.

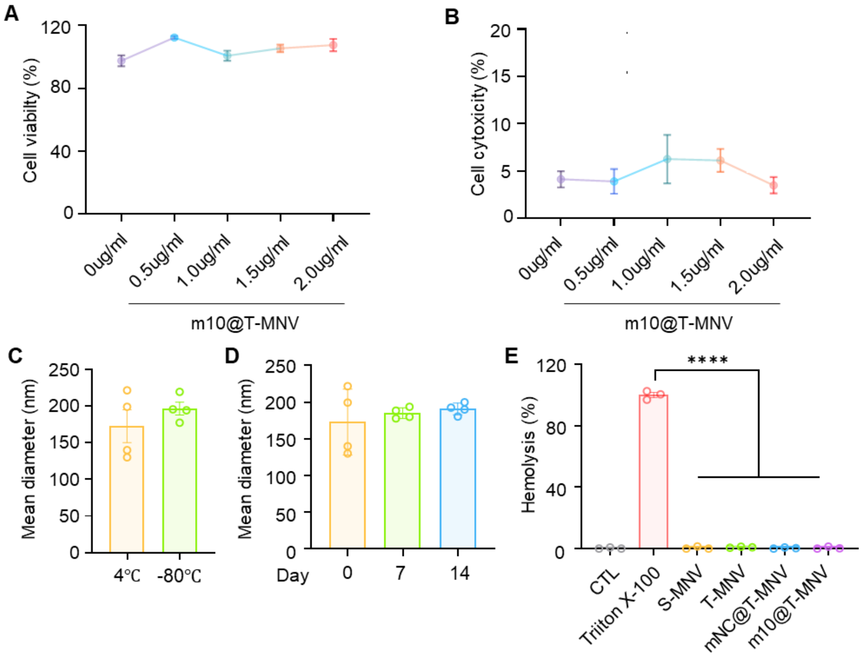

Figure S7. Evaluation of biocompatibility, stability, and hemocompatibility of *m10*@T-MNVs. A) Cell viability of H9C2 cardiomyocytes treated with increasing concentrations of *m10*@T-MNVs. B) Lactate dehydrogenase (LDH) release assay for assessing cytotoxicity in H9C2 cells after *m10*@T-MNVs treatment. C) Measurement of the mean diameter of *m10*@T-MNVs under different storage conditions (4 °C and -80° C). D) Measurement of the mean diameter of different time points (Day 0, 7, and 14), showing stability over time. E) Hemolysis assays show the hemolytic activity of various groups compared to the control. Statistical significance was determined using one-way ANOVA with Tukey's post-hoc test. Data are presented as mean ± SD. ns, not significant; ****$p < 0.0001$.

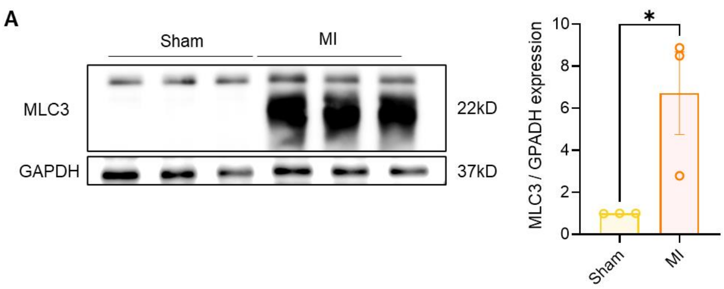

Figure S8. MLC3 expression in infarcted myocardium. A) Representative Western blot images and quantification of MLC3 expression in sham and myocardial infarction (MI) heart tissues. MLC3 signals were normalized to GAPDH and expressed relative to the sham group. Each data point represents an individual animal ($n = 3$). Statistical significance was determined using unpaired two-tailed Student's *t*-test. Data are presented as mean ± SD. $*p < 0.05$.

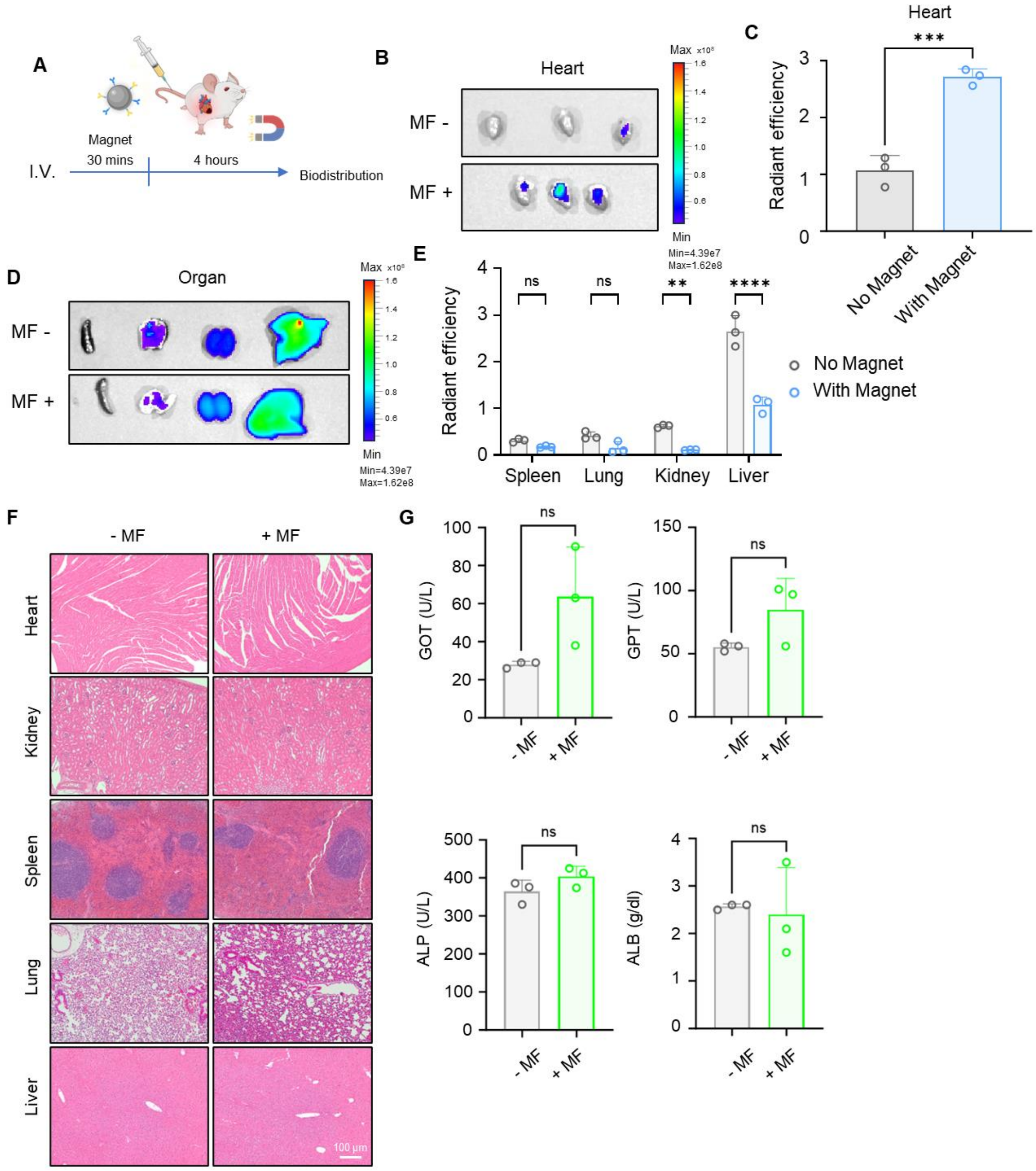

Figure S9. Magnetic targeting and biodistribution analysis of IONP-Abs *in vivo.* A) Schematic showing intravenous (I.V.) administration of IONP-Abs followed by magnetic exposure over the chest for 30 min and organ harvesting 4 h post-injection. B) IVIS imaging of harvested hearts from mice treated with or without magnetic field (MF). C) Quantification of radiant efficiency in the heart with and without MF application. D) Ex vivo fluorescence images of major organs (spleen, lung, kidney, liver) comparing biodistribution with and without MF. E) Quantified radiant efficiency values in individual organs following *m10*@T-MNVs administration. F) Representative H&E-stained sections of heart, kidney, spleen, lung, and liver to evaluate histological toxicity. Scale bar: 100 µm. G) Systemic toxicity analysis of liver and

kidney function markers (GOT, GPT, ALP, ALB) in mice treated with or without MF. Statistical significance was determined using one-way ANOVA with Tukey's post-hoc test. Data are presented as mean ± SD. ns, not significant; $^{**}p < 0.01$, $^{***}p < 0.001$, and $^{****}p < 0.0001$.

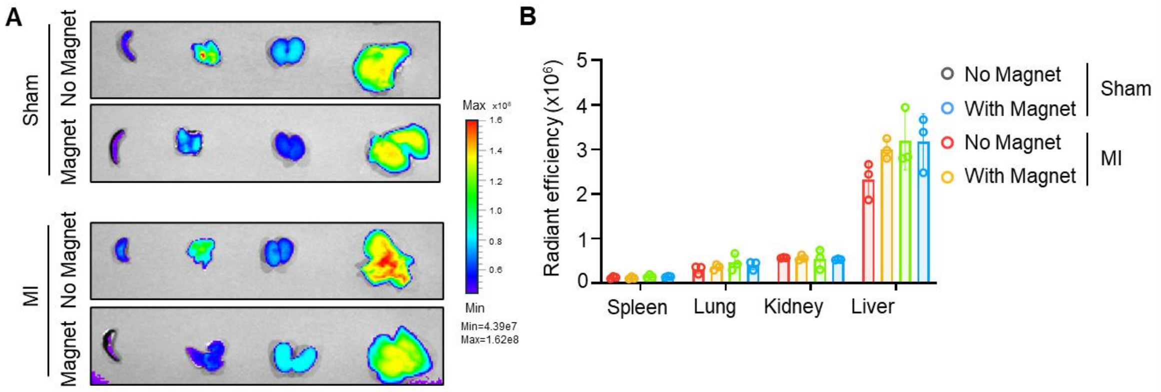

Figure S10. Evaluation of biodistribution of *m10*@T-MNVs in myocardial infarction (MI) and sham mice. A) IVIS imaging of major organs (spleen, lung, kidney, and liver) from sham and MI mice, with or without magnetic field application following intravenous administration of *m10*@T-MNVs. B) Quantitative analysis of radiant efficiency across organs from sham and MI mice, in the presence or absence of magnetic targeting. Statistical significance was determined using one-way ANOVA with Tukey's post-hoc test. Data are presented as mean ± SD. ns, not significant.

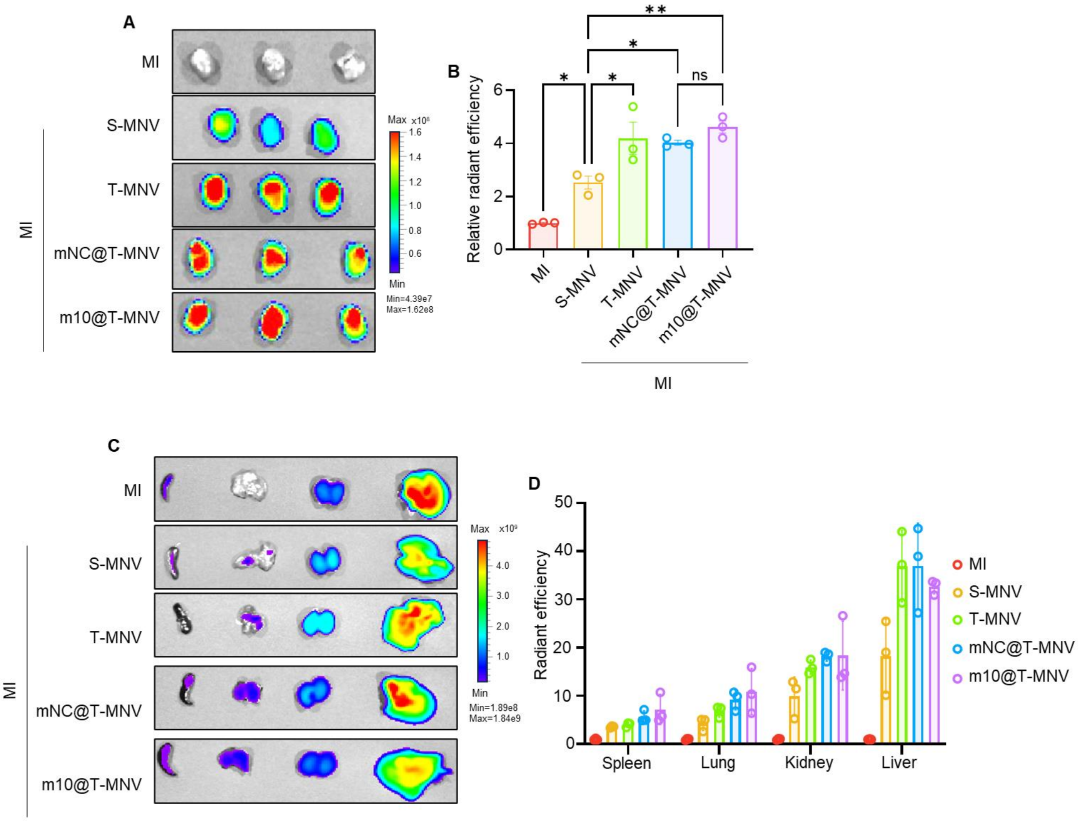

Figure S11. Comparative biodistribution and cardiac accumulation of targeted and non-targeted magnetic nanovesicles in myocardial infarction (MI) mice. A) IVIS images of harvested hearts from MI mice intravenously injected with different formulations, including MI, S-MNVs, T-MNVs, *mNC*@T-MNVs, and *m10*@T-MNVs. B) Quantitative analysis of relative radiant efficiency in the heart across treatment groups. C) Fluorescence imaging of major organs (spleen, lung, kidney, liver) following administration of the indicated nanovesicle formulations. D) Quantified radiant efficiency in individual organs reflecting biodistribution profiles of the tested nanovesicles. Statistical significance was determined using one-way ANOVA with Tukey's post-hoc test. Data are presented as mean ± SD. ns, not significant; $*p < 0.05$, $**p < 0.01$.

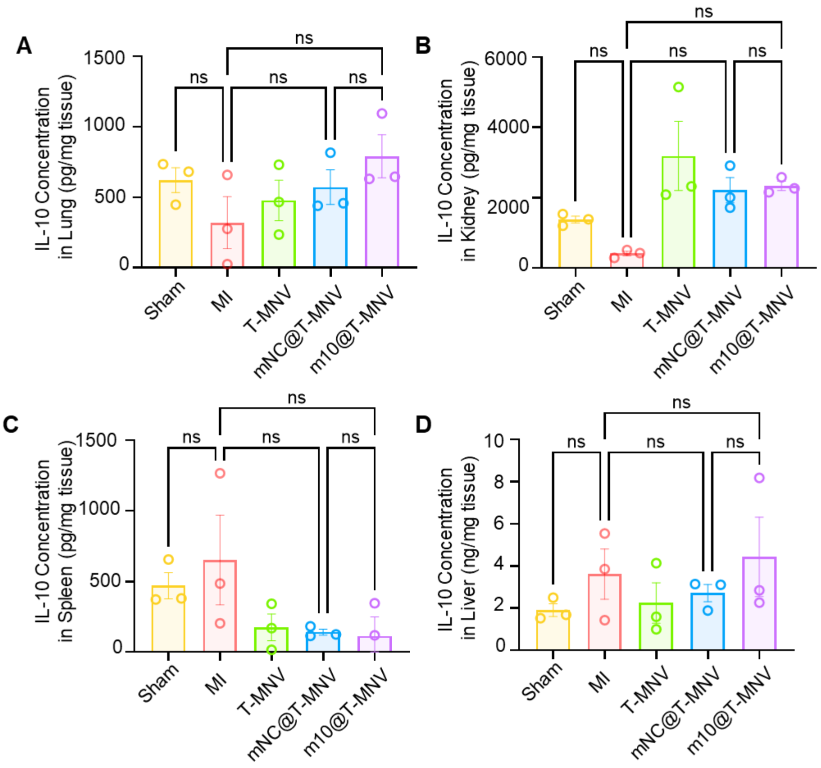

Figure S12. Quantification of IL-10 accumulation in major organs after systemic administration of magnetic nanovesicles. A-D) IL-10 protein concentration in tissue collected from sham and MI mice treated with T-MNVs, *mNC*@T-MNVs, or *m10*@T-MNVs measured by ELISA in A) Lung, B) Kidney, C) Spleen, and D) Liver. Statistical significance was determined using one-way ANOVA with Tukey's post-hoc test. Data are presented as mean ± SD. ns, not significant.

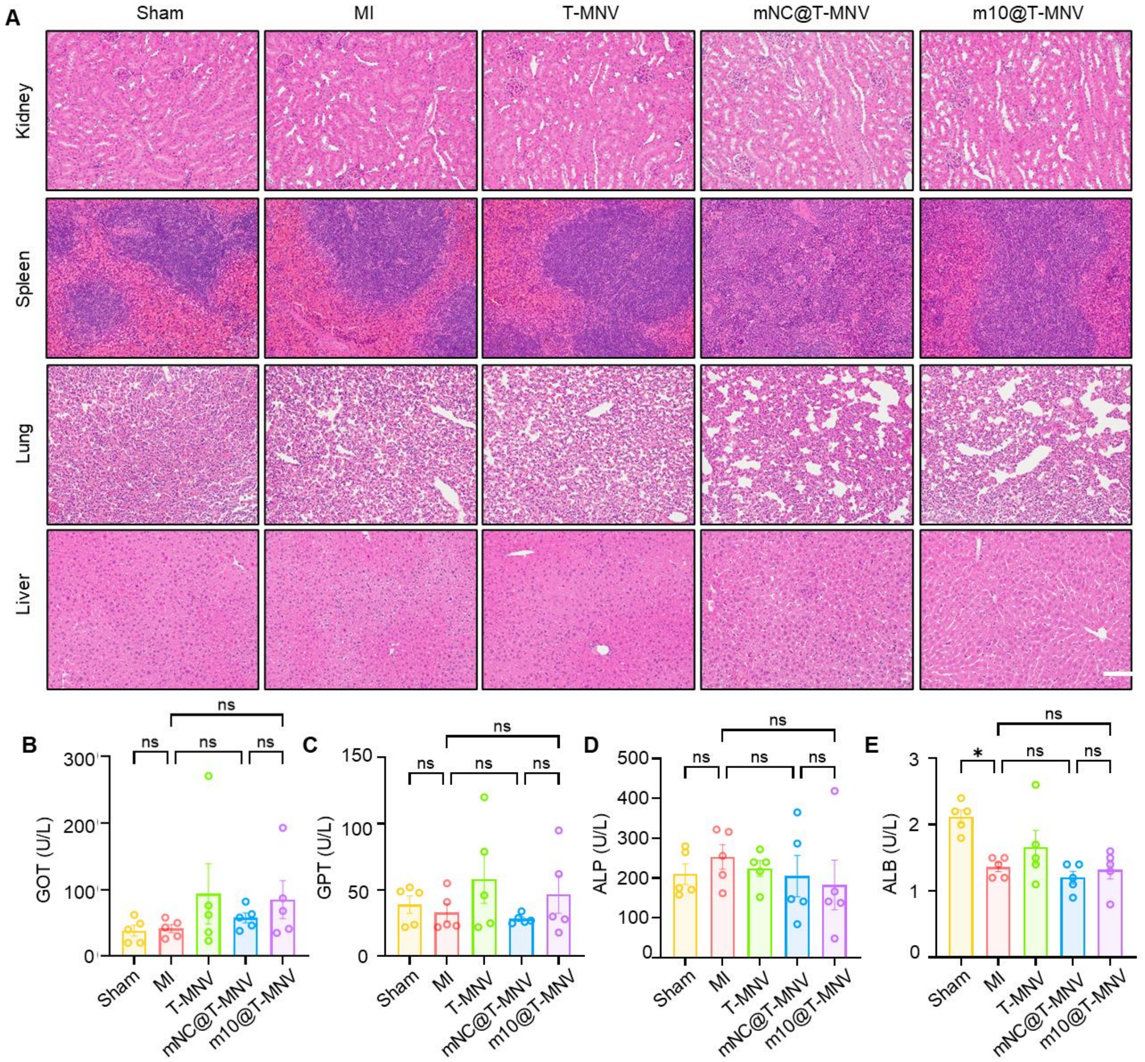

Figure S13. Histological and serological evaluation of systemic toxicity following administration of *m10*@T-MNVs. A) Representative hematoxylin and eosin (H&E)-stained images of kidney, spleen, lung, and liver tissues collected from sham, MI, and treated mice (T-MNVs, *mNC*@T-MNVs, *m10*@T-MNVs). Scale bar: 100 μm. B-E) Serum biochemical evaluation of systemic toxicity via quantification of liver function biomarkers, including B) GOT, C) GPT, D) ALP, and E) ALB. Statistical significance was determined using one-way ANOVA with Tukey's post-hoc test. Data are presented as mean ± SD. ns, not significant; $*p < 0.05$.

Table S1. mRNA sequence

**IL-10 mRNA**	ATGCCTGGCTCAGCACTGCTATGCTGCCTGCTCTTACTGACTGGCATGAGG<br>ATCAGCAGGGGCCAGTACAGCCGGGAAGACAATAACTGCACCCACTTCCC<br>AGTCGGCCAGAGCCACATGCTCCTAGAGCTGCGGACTGCCTTCAGCCAGGT<br>GAAGACTTTCTTTCAAACAAAGGACCAGCTGGACAACATACTGCTAACCGA<br>CTCCTTAATGCAGGACTTTAAGGGTTACTTGGGTTGCCAAGCCTTATCGGA<br>AATGATCCAGTTTTACCTGGTAGAAGTGATGCCCCAGGCAGAGAAGCATGG<br>CCCAGAAATCAAGGAGCATTTGAATTCCCTGGGTGAGAAGCTGAAGACCCT<br>CAGGATGCGGCTGAGGCGCTGTCATCGATTTCTCCCCTGTGAAAATAAGAG<br>CAAGGCAGTGGAGCAGGTGAAGAGTGATTTTAATAAGCTCCAAGACCAAG<br>GTGTCTACAAGGCCATGAATGAATTTGACATCTTCATCAACTGCATAGAAG<br>CATACATGATGATCAAAATGAAAAGCTAA

Table S2. Primer sequences for quantitative real-time PCR.

Gene	Forward Primer	Reverse Primer
**GAPDH**	CCA TAG CTG AAC TGA AAA CCA CC	GGT GGT CCA GGG TTT CTT ACT
**IL-10**	TCT CCG AGA TGC CTT CAG CAG A	TCA GAC AAG GCT TGG CAA CCC A
**iNOS**	ACA TCG ACC CGT CCA CAG TAT	CAG AGG GGT AGG CTT GTC TC
**TNF-α**	CAG GCG GTG CCT ATG TCT C	CGA TCA CCC CGA AGT TCA GTA G
**Arg1**	CTC CAA GCC AAA GTC CTT AGA G	GGA GCT GTC ATT AGG GAC ATC A
**TGF-ß**	TTG TTG CCC TCC TAC AGA CTG G	GTA AAG AGG GCG AAG GCA GCA A
